# Radixin regulates synaptic GABA_A_ receptor density and is essential for reversal learning and short-term memory

**DOI:** 10.1038/ncomms7872

**Published:** 2015-04-20

**Authors:** Torben J. Hausrat, Mary Muhia, Kimberly Gerrow, Philip Thomas, Wiebke Hirdes, Sachiko Tsukita, Frank F. Heisler, Lena Herich, Sylvain Dubroqua, Petra Breiden, Joram Feldon, Jürgen R Schwarz, Benjamin K. Yee, Trevor G. Smart, Antoine Triller, Matthias Kneussel

**Affiliations:** 1 University Medical Center Hamburg-Eppendorf, Center for Molecular Neurobiology, ZMNH, Institut for Molecular Neurogenetics, Falkenried 94, 20251 Hamburg, Germany; 2 Biologie Cellulaire de la Synapse, Ecole Normale Supérieure, Inserm U1024, CNRS, UMR8197, PSL Research University, 46 rue d'Ulm, 75005 Paris, France; 3 University College London, Neuroscience, Physiology & Pharmacology, Gower Street, WC1E6BT London, UK; 4 Osaka University, Laboratory of Biological Science, Graduate School of Frontier Bioscience and Graduate School of Medicine, Yamadaoka 2-2, Suita, Osaka 565-0871, Japan; 5 University Medical Center Hamburg-Eppendorf, Medical Biometry and Epidemiology, Martinistraße 52, 20246 Hamburg, Germany; 6 Swiss Federal Institute of Technology Zurich, Behavioural Neurobiology, Schorenstrasse 16, 8603 Schwerzenbach, Switzerland

## Abstract

Neurotransmitter receptor density is a major variable in regulating synaptic strength. Receptors rapidly exchange between synapses and intracellular storage pools through endocytic recycling. In addition, lateral diffusion and confinement exchanges surface membrane receptors between synaptic and extrasynaptic sites. However, the signals that regulate this transition are currently unknown. GABA_A_ receptors containing α5-subunits (GABA_A_R-α5) concentrate extrasynaptically through radixin (Rdx)-mediated anchorage at the actin cytoskeleton. Here we report a novel mechanism that regulates adjustable plasma membrane receptor pools in the control of synaptic receptor density. RhoA/ROCK signalling regulates an activity-dependent Rdx phosphorylation switch that uncouples GABA_A_R-α5 from its extrasynaptic anchor, thereby enriching synaptic receptor numbers. Thus, the unphosphorylated form of Rdx alters mIPSCs. *Rdx* gene knockout impairs reversal learning and short-term memory, and Rdx phosphorylation in wild-type mice exhibits experience-dependent changes when exposed to novel environments. Our data suggest an additional mode of synaptic plasticity, in which extrasynaptic receptor reservoirs supply synaptic GABA_A_Rs.

## Abstract (web summary)

Lateral diffusion of receptors between synaptic and extrasynaptic sites is known to mediate plasticity. Hausrat *et al*. show that diffusion of α5-containing GABA_A_ receptors is controlled by phosphorylation of the extrasynaptic anchoring protein Radixin, and reveal a role for Radixin in learning and memory.

## Graphical abstract

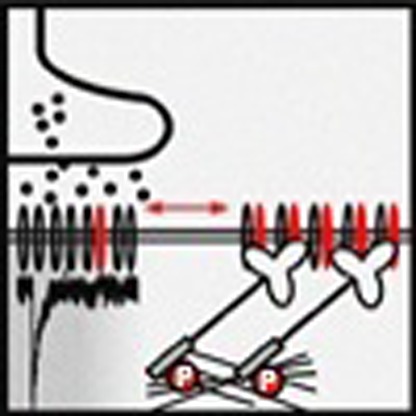

Synaptic plasticity, the ability of synapses to change their strength, is a critical feature in learning and memory processing^1^. Synaptic strength depends highly on neurotransmitter receptor density and turnover at post-synaptic sites. Mechanistically, receptor turnover employs two main trafficking modes to modulate the concentration of receptors at synapses. First, exo- and endocytic processes exchange vesicular receptors between cytosolic compartments and the plasma membrane^2^,^3^. Endocytic recycling of α-amino-3-hydroxy-5-methyl-4-isoxazolepropionic acid (AMPA) receptors from intracellular storage pools is an efficient mechanism for the recruitment of receptors to post-synaptic sites during long-term potentiation (LTP)^4^. Second, lateral diffusion participates in the exchange of cell surface receptors between synaptic and extrasynaptic domains^5^. Although diffusion is non-directional, submembrane scaffolding proteins act as affinity traps to confine and concentrate diffusing receptors not only at synapses, but also at extrasynaptic sites^6^,^7^,^8^,^9^.

The post-synaptic scaffolding protein gephyrin localizes at inhibitory synapses and binds α1, α2, α3, β2 and β3 subunit-containing GABA_A_ receptors (GABA_A_Rs)^10^,^11^. Gephyrin is crucial for receptor clustering at GABAergic synapses^12^,^13^. In contrast, the actin-binding protein radixin (Rdx), an ezrin/radixin/moesin (ERM)-family member^14^, specifically anchors GABA_A_R-α5 at extrasynaptic sites^7^. To achieve this, Rdx requires a two-step activation process, mediated through membrane association and a phosphorylation-dependent conformational change. The latter is thought to be RhoA GTP- and Rho-kinase (ROCK) dependent^15^.

GABA_A_R-α5 is mainly expressed in the hippocampus and is implicated in hippocampal-dependent learning processes^16^. Although GABA_A_R-α5 receptors are reported to participate in phasic inhibition at synapses and mediate a slowly decaying inhibitory synaptic current^17^,^18^, their precise physiological function currently remains unclear. The majority of GABA_A_R-α5 receptors localize extrasynaptically and mediate tonic inhibition, a critical feature in the control of neuronal excitability, learning and memory^19^,^20^. However, it remains to be determined: (i) why extrasynaptic GABA_A_R-α5 surface membrane receptors accumulate extrasynaptically to synapses^7^,^21^,^22^ and (ii) which parameters regulate the synaptic versus extrasynaptic localization of this receptor subtype.

Here we report that Rdx-mediated extrasynaptic surface membrane clusters of GABA_A_R-α5 constitute potential reservoirs to rapidly supply GABA_A_R-α5 receptors to inhibitory synapses in an activity-dependent manner. Furthermore, Rdx depletion in mice impairs specific forms of learning and memory.

## Results

### Rdx dephosphorylation increases synaptic GABA_A_R-α5

**Figure 1:**
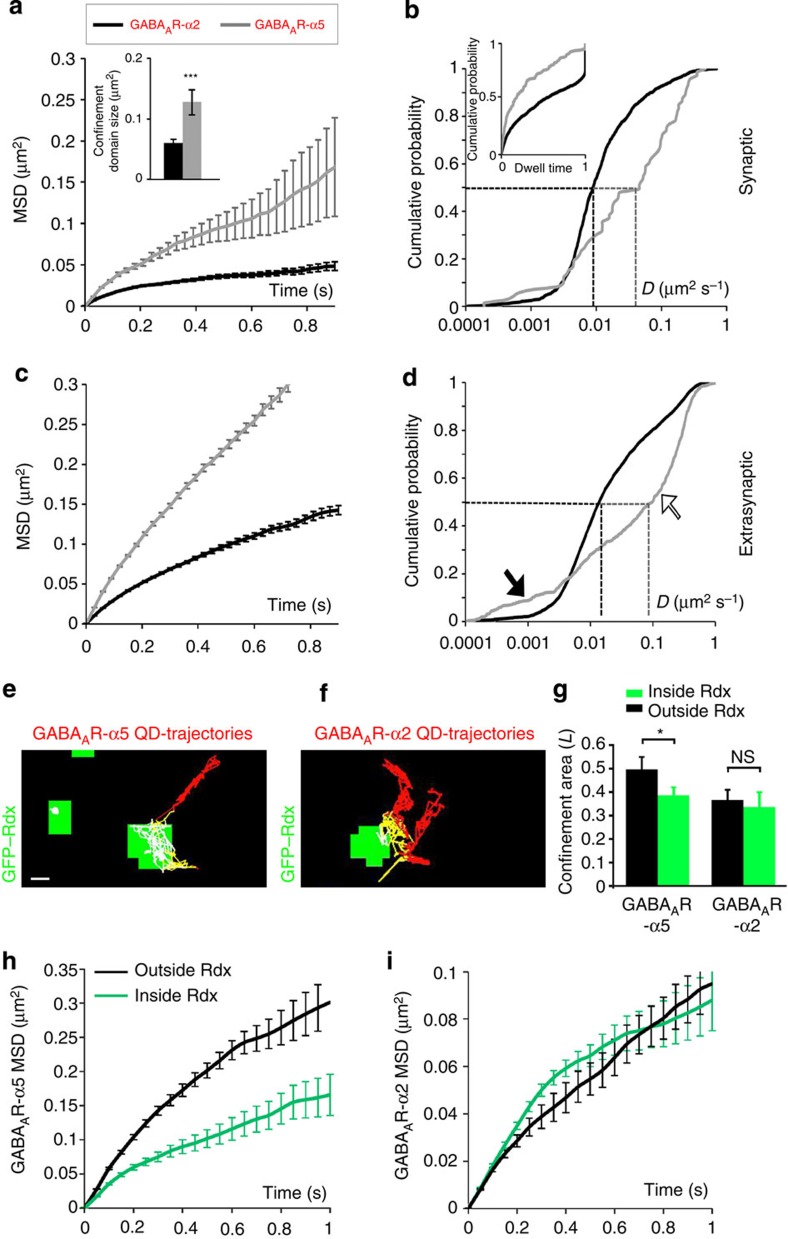
Diffusion properties of GABA_A_R-α2 and GABA_A_R-α5 inside and outside of GFP–Rdx clusters. (**a**) Mean square displacement (MSD) versus time plot for GABA_A_ receptors containing α2 subunits (black) and GABA_A_ receptors containing α5 subunits (grey) quantum dot (QD) trajectories at gephyrin-positive inhibitory synapses. Inset: average size of the confinement domain for GABA_A_R-α2 (black, 0.059±0.006 μm^2^) versus GABA_A_R-α5 (grey, 0.127±0.020 μm^2^, *P*<0.001, *t*-test) at synapses. (**b**) Cumulative probability plot for GABA_A_R-α2- and GABA_A_R-α5-QD diffusion coefficients at gephyrin-positive inhibitory synapses (*P*<0.001, Kolmogorov-Smirnov (KS) test). Median diffusion coefficients for GABA_A_R-α2 (0.009 μm^2^ s^−1^) and GABA_A_R-α5 (0.046 μm^2^ s^−1^) are indicated by dashed lines. Inset: cumulative probability of dwell time (time at synapses/total time) at synapses (*P*<0.001, KS test). (**c**) MSD versus time plot for GABA_A_R-α2 (black) and GABA_A_R-α5 (grey) QD trajectories at extrasynaptic sites. (**d**) Cumulative probability plot of GABA_A_R-α2 and GABA_A_R-α5 QD diffusion coefficients at extrasynaptic sites (*P*<0.001, KS test). Median diffusion coefficients for GABA_A_R-α2 (0.014 μm^2^ s^−1^) and GABA_A_R-α5 (0.097 μm^2^ s^−1^) are indicated by dashed lines. Solid arrow: population of extrasynaptic GABA_A_R-α5 with a smaller diffusion coefficient than GABA_A_R-α2. Open arrow: population of extrasynaptic GABA_A_R-α5 with a greater diffusion coefficient than GABA_A_R-α2. (**e**,**f**) Representative extrasynaptic QD trajectories of (**e**) GABA_A_R-α5 and (**f**) GABA_A_R-α2 in hippocampal neurons expressing GFP–Rdx (green). Receptor trajectories: outside of Rdx cluster (red), peripheral (yellow), inside clusters (white). (**g**) Confinement area of GABA_A_R-α5 and GABA_A_R-α2 inside (α2: 0.33±0.06; α5: 0.38±0.03) and outside (α2: 0.36±0.04; α5: 0.49±0.05) of Rdx cluster (*n*=59/78, *P*<0.05, paired *t*-test). (**h**,**i**) MSD versus time plot for (**h**) GABA_A_R-α5- and (**i**) GABA_A_R-α2-QD trajectories outside (black) and inside (green) of Rdx-positive cluster at extrasynaptic sites. Scale bars, 500 nm. Error bars, mean±s.e.m. NS, not significant.

To analyse whether GABA_A_R-α5 receptors generally exchange between synaptic and extrasynaptic plasma membrane positions, we applied single particle tracking (SPT) using quantum dot (QD) imaging^5^,^23^. Receptors entered and left synaptic areas, with GABA_A_R-α5 displaying significantly less confinement at inhibitory synapses compared with synaptically enriched receptors containing GABA_A_R-α2 (Fig. 1a,b) or GABA_A_R-α1 (Supplementary Fig. 1a,b). Notably, two populations of diffusing GABA_A_R-α5 were identified outside synapses: one with higher and one with lower diffusion coefficients compared with GABA_A_R-α2 (Fig. 1c,d, arrows) or GABA_A_R-α1 (Supplementary Fig. 1c,d, arrows). These data indicate the presence of freely mobile, as well as affinity-trapped extrasynaptic receptors. Since the latter has been shown to interact with the GABA_A_R-α5 binding protein Rdx^7^, we also analysed GABA_A_R-α5 diffusion probabilities inside and outside of Rdx-positive sites. Accordingly, the diffusion coefficient for GABA_A_R-α5, but not for GABA_A_R-α2, was significantly reduced at Rdx clusters that mainly locate extrasynaptically (Fig. 1e–i).

**Figure 2:**
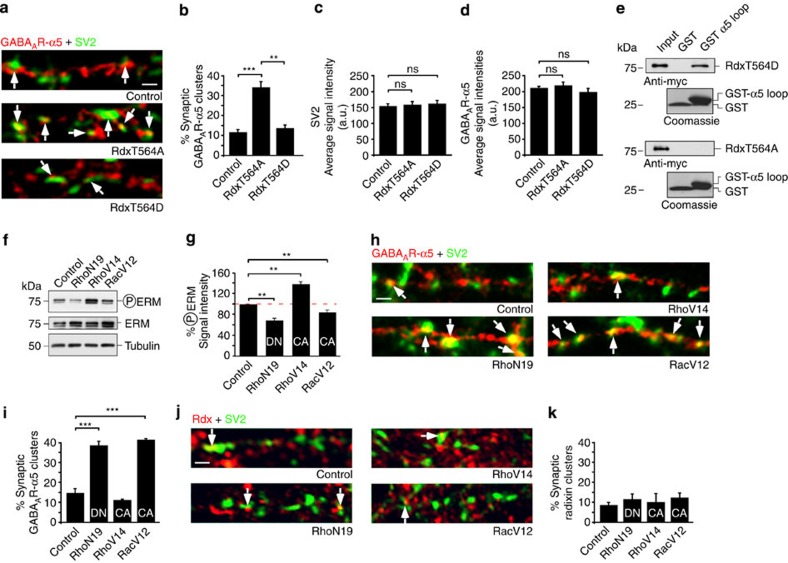
RhoA GTPase-dependent Rdx dephosphorylation increases GABA_A_R-α5 synaptic density. Arrows: synaptic co-localization of endogenous GABA_A_R-α5 or radixin (Rdx) with the pre-synaptic vesicle marker SV2. (**a**) Dendritic regions of neurons expressing RdxT564A or RdxT564D compared with neurons expressing control GFP. (**b**) RdxT564A causes significant synaptic GABA_A_R-α5 enrichment (34.20±5.17%; 3.06±0.42 cluster per 20 μm dendrite) compared with RdxT564D (13.78±2.84%; 1.28±0.23 cluster per 20 μm dendrite) or with control (11.66±2.53%; 1.08±0.21 cluster per 20 μm dendrite) (compare with Fig. 9) (*n*=3; 10–16 cells per experiment). (**c**,**d**) Signal intensities remained unchanged for SV2 (control: 152.06±6.43 a.u., RdxT564A: 156.32±8.51 a.u., RdxT564D: 159.97±8.51 a.u.) and GABA_A_R-α5 (control: 208.29±4.20 a.u., RdxT564A: 216.32±9.35 a.u., RdxT564D: 195.91±10.45 a.u.). (**e**) Glutathione *S*-transferase (GST) pull-down assays detect binding of RdxT564D, but no binding of RdxT564A to the GABA_A_R-α5 cytosolic loop. (**f**,**g**) Phosphorylated-ERM (PERM) levels following heterologous expression of constitutively active (CA) or dominant-negative (DN) Rho-GTPase mutants in HEK293 cells. RhoN19 (DN) (68.41±3.14%) and RacV12 (CA) (83.94±4.25%) decrease phosphorylation. Conversely, RhoV14 (CA) (137.54±4.76%) significantly increases phosphorylation (*n*=6). (**h**,**i**) Synaptic localization of GABA_A_R-α5 in dendrites from neurons expressing Rho-GTPase mutants. Note: decreased phosphorylation levels inversely correlate with increased GABA_A_R-α5 at synapses (see Fig. 2f,g) (control: 14.89±2.30%, RhoN19: 38.67±2.41%, RhoV14: 11.21±0.49%, RacV12: 41.43±0.93%) (*n*=3; 10–16 cells per experiment). (**j**,**k**) Synaptic localization of Rdx in dendrites from neurons expressing Rho-GTPase mutants. Quantification of Rdx immuno-reactive signals at synaptic sites reveals no significant differences on expression of RhoA GTPase mutants (control: 8.55±1.40%, RhoN19: 11.59±3.07%, RhoV14: 10.20±4.60%, RacV12: 12.29±2.45%) (*n*=3; 9–11 cells per experiments). Error bars, mean±s.e.m. Scale bars, 3 μm. ANOVA and the one-sample *t*-test against 100% were used for statistical analysis (***P<0.001; **P<0.01). NS, not significant.

To examine whether Rdx plays a role in regulating synaptic versus extrasynaptic GABA_A_R-α5 receptors, we overexpressed a Rdx point mutant (RdxT564A) in hippocampal neurons. This mutant prevents threonine phosphorylation, which is critical for activating Rdx via a two-step intramolecular conformational change^15^,^24^. Consequently, RdxT564A cannot be phosphorylated, but remains localized at the plasma membrane (Supplementary Fig. 2d–g) thereby displacing endogenous Rdx. RdxT564A overexpression tripled the density of GABA_A_R-α5 at SV2-positive synapses, compared with ∼12% synaptic GABA_A_R-α5 observed under control conditions (Fig. 2a,b and Supplementary Fig. 2h). This observation was specific given that a phospho-mimetic constitutively active (CA) Rdx mutant (RdxT564D) induced no such effect (Fig. 2a,b and Supplementary Fig. 2c,e–h). Moreover, neither RdxT564A nor RdxT564D overexpression affected SV2 and GABA_A_R-α5 average signal intensities or the density of gephyrin-positive inhibitory synapses (Fig. 2c,d and Supplementary Fig. 2i–k). These data suggested that a phosphorylation-minus mutant of Rdx (RdxT564A) increases the amount of synaptic GABA_A_R-α5 accumulation. Consistent with this, the GABA_A_R-α5 intracellular loop specifically bound to the phospho-mimetic form of Rdx (RdxT564D), but failed to bind unphosphorylated Rdx (RdxT564A) (Fig. 2e and Supplementary Fig. 2l), suggesting that GABA_A_R-α5 receptors can enter synaptic domains when devoid of their anchoring factor Rdx.

**Figure 3:**
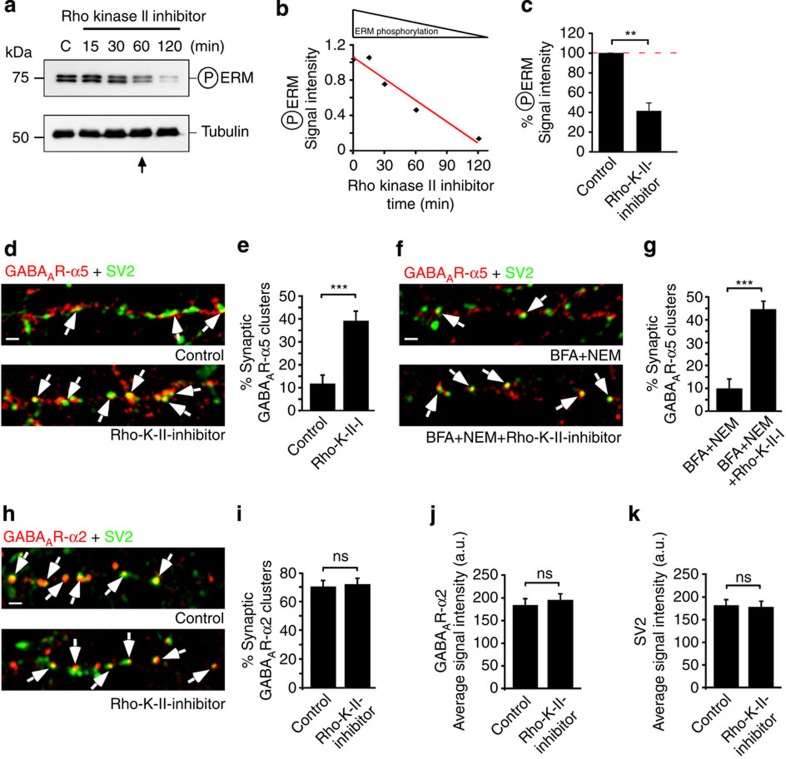
Rho kinase inhibition decreases PERM levels and increases GABA_A_R-α5 synaptic concentration. (**a**,**b**) Rho kinase II inhibitor decreases phosphorylated ezrin/radixin/moesin (PERM) levels in hippocampal neurons over time. (**c**) Quantification of PERM levels on Rho kinase II inhibitor treatment for 1 h (control: 100%, Rho-K-II-inhibitor: 40.30±7.80%; graph corresponds to 60 min values shown in **a** (black arrow); *n*=4). (**d**–**g**) Synaptic localization of GABA_A_R-α5 in neuronal dendrites (white arrows) treated with Rho kinase II inhibitor in the (**d**) absence and (**f**) presence of BFA and NEM for 1 h. Quantification of *n*=3 experiments with 12–20 cells each. Note: decreased phosphorylation of Rdx through Rho kinase II inhibitor increases synaptic GABA_A_R-α5 levels (see **e**; control: 11.59±3.71%, Rho-K-II-inhibitor: 38.90±4.04%) also in the presence of BFA and NEM (see **g**; BFA+NEM: 9.82±4.04%, BFA+NEM+Rho-K-II-inhibitor: 44.27±3.71%). (**h**,**i**) Synaptic localization of GABA_A_R-α2 (control: 70.69±3.90%, Rho-K-II-inhibitor: 71.55±3.90%) and signal intensities for (**j**) GABA_A_R-α2 (control: 181.03±13.23%, Rho-K-II-inhibitor: 192.02±13.23%) and (**k**) SV2 (control: 178.43±11.14%, Rho-K-II-inhibitor: 174.86±11.14%) remain unchanged after Rho-K-II-inhibitor treatment indicating no increase in synapse density. Error bars, mean±s.e.m. Scale bars, 3 μm. The Student's *t*-test and ANOVA were used for statistical analysis (***P<0.001; **P<0.01). NS, not significant.

Previous studies have suggested that RhoA GTPases activate Rdx through T564 phosphorylation^15^ (Supplementary Fig. 2a). We therefore used dominant-negative (DN, RhoN19) or CA (RhoV14) mutants of RhoA, or a CA mutant of the RhoA counterplayer Rac1 (RacV12)^25^, and assessed Rdx phosphorylation levels. Indeed, RhoN19 (DN) and RacV12 (CA) significantly reduced Rdx phosphorylation levels, which were detected with an ERM-phosphospecific antibody^26^ that recognizes the conserved T564 residue (Fig. 2f,g). In contrast, CA RhoA (RhoV14) increased phosphorylated-ERM (PERM) levels. Consistent with our previous observation (unphosphorylatable RdxT564A; Fig. 2a,b), lower Rdx phosphorylation levels induced by DN RhoN19 or CA RacV12 were correlated with higher concentrations of GABA_A_R-α5 at synapses (Fig. 2h,i and Supplementary Fig. 2m). This suggested that lower levels of phosphorylated Rdx (Fig. 2f,g) increase the amount of synaptic GABA_A_R-α5 accumulation. Conversely, increased Rdx phosphorylation via CA RhoV14 confined GABA_A_R-α5 to extrasynaptic sites (Fig. 2h,i, and Supplementary Fig. 2m). Notably, GTPase mutants exclusively altered receptor localization but did not alter the extrasynaptic localization of Rdx (Fig. 2j,k, and Supplementary Fig. 2n). To further support that RhoA-dependent Rdx phosphorylation influences GABA_A_R-α5 localization, we applied Rho kinase II inhibitor^27^ to hippocampal neurons. The drug gradually decreased PERM levels over time (Fig. 3a–c) and consistently elevated the synaptic GABA_A_R-α5 concentration (Fig. 3d,e). This observation appears to be independent of Golgi vesicle segregation and exocytosis, since brefeldin A and N-ethylmaleimide did not influence the effect (Fig. 3f,g and Supplementary Fig. 3a–d). Control experiments revealed that Rho kinase II inhibitor did not affect GABA_A_R-α2 synaptic localization, receptor signal intensity or pre-synaptic SV2 signals (Fig. 3h–k). We therefore hypothesized that unphosphorylated Rdx, regulated through RhoA signalling, might be a prerequisite for GABA_A_R-α5 uncoupling from its extrasynaptic anchor, thereby promoting post-synaptic receptor entry.

### Rdx depletion increases synaptic GABA_A_R-α5 cluster sizes

**Figure 4:**
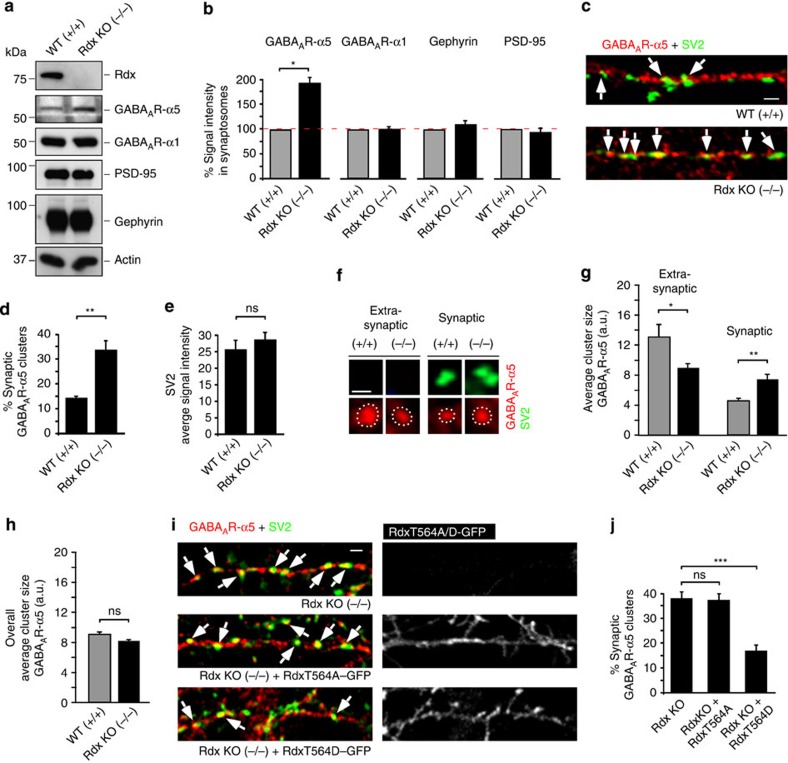
Rdx depletion in mice increases GABA_A_R-α5 synaptic concentration and cluster size that is reversible through RdxT564D expression. (**a**) GABA_A_R-α5 is specifically and significantly enriched in synaptosomes from Rdx-depleted (−/−) compared with WT control (+/+) mouse forebrain lysates. (**b**) Quantification of three independent experiments. Note: GABA_A_R-α5 levels are increased in synaptosomes derived from *Rdx* (−/−) (173.88±14.10%) mice, whereas GABA_A_R-α1 (99.39±2.63%), gephyrin (112.60±6.86%) and PSD-95 (94.74±8.51%) levels remain unaltered compared with WT (+/+) controls (set to 100%). (**c**) Synaptic localization of GABA_A_R-α5 in dendrites (arrows) of hippocampal neurons derived from WT (+/+) and *Rdx* (−/−) mice. (**d**,**e**) Rdx depletion causes significant enrichment of GABA_A_R-α5 in apposition to SV2-positive pre-synaptic terminals ((+/+): 14.29±0.38%, (−/−): 33.60±3.44%). However, it does not alter SV2 average signal intensities ((+/+): 25.53±2.79, (−/−): 28.51±2.07) (*n*=3 with 5–14 cells per experiment). (**f**) Representative GABA_A_R-α5 cluster sizes at synaptic and extrasynaptic sites in hippocampal neurons from WT (+/+) and *Rdx* (−/−) mice. (**g**,**h**) Analysis of GABA_A_R-α5 cluster sizes revealed enlarged synaptic clusters ((+/+): 4.57±0.36 a.u., (−/−): 7.40±0.23 a.u.) accompanied by decreased extrasynaptic (SV2 negative) clusters on Rdx depletion ((+/+): 13.07±0.32 a.u., (−/−): 8.98±0.25 a.u.). Notably, the quantification of pooled synaptic and extrasynaptic cluster size (**h**) reveals no significant difference between both genotypes ((+/+): 9.05±0.22 a.u., (−/−): 8.16±0.16 a.u.). (**i**,**j**) Synaptic localization of GABA_A_R-α5 in dendrites (arrows) of hippocampal neurons derived from *Rdx* (−/−) after expression of RdxT564A–GFP (37.43±2.60%) and RdxT564D–GFP (16.96±2.79%), as compared with non-GFP-expressing cells (38.08±2.69%) of the same culture (see grey panels in **i** for GFP signals). Error bars, mean±s.e.m. Scale bars, (**c**,**i**) 3 μm, (**f**) 1 μm. The Student's *t*-test (**b**,**d**,**e**) or ANOVA (**g**,**h**,**j**) was used for statistical analysis (**P<0.01; *P<0.05). NS, not significant.

Based on these observations, we expected that *Rdx* knockout (KO) mice^28^ would show higher GABA_A_R-α5 concentrations at synapses *in vivo*. Adult mice synaptosomal fractions containing high levels of the excitatory (post-synaptic density-95 (PSD-95)) and inhibitory (gephyrin) synapse markers^29^ supported this view. Compared with actin loading controls, GABA_A_R-α5 was significantly enriched in Rdx-depleted (−/−) synaptosomal fractions (Fig. 4a,b) while another GABA_A_ receptor subunit (GABA_A_R-α1), as well as pre- and post-synaptic marker proteins remained unchanged (Supplementary Fig. 4a–c). Additional, quantitative immunocytochemistry confirmed enriched GABA_A_R-α5 signals in (−/−) neurons in apposition to SV2-positive terminal boutons (Fig. 4c,d, and Supplementary Fig. 4d). SV2 signal intensities were comparable between genotypes (Fig. 4e), which indicated that there was no increase in synapse density. This led us to conclude that GABA_A_R-α5 can redistribute into synapses in the absence of its extrasynaptic membrane anchor Rdx.

To distinguish synaptic from extrasynaptic locations, we quantified pixel areas of GABA_A_R-α5 cluster sizes that were either apposed or non-apposed to the pre-synaptic vesicle marker SV2. Contrary to a previous study using hippocampal cryosections^7^, GABA_A_R-α5 extrasynaptic clusters were not completely lost, but were significantly reduced. Extrasynaptic GABA_A_R-α5 clusters in wild-type (WT) neurons (+/+) were about three times larger than their synaptic counterparts (Fig. 4f,g). In Rdx-deficient neurons (−/−) a significant increase in synaptic GABA_A_R-α5 cluster size was observed (Fig. 4f,g, and Supplementary Fig. 4e), indicating that Rdx functions as an anchor to retain GABA_A_R-α5 extrasynaptically. Importantly, a combined size analysis of all receptor clusters (synaptic and extrasynaptic) was highly similar between WT and Rdx-depleted neurons (Fig. 4h). Thus, the observed changes in receptor cluster size (Fig. 4g) are likely to represent a re-distribution of receptors between extrasynaptic and synaptic sites within the plasma membrane.

To analyze whether RdxT564 phosphorylation is the critical parameter for the observed receptor re-distribution, we overexpressed the phopho-minus (RdxT564A) and phospho-mimicking (RdxT564D) mutant in neurons derived from *Rdx*-KO mice. Overexpression of RdxT564D, but not RdxT564A, reversed the increase in synaptic GABA_A_R-α5 localization found in (−/−) neurons (Fig. 4i,j), indicating that Rdx phosphorylation, mimicked through RdxT564D, is a prerequisite for extrasynaptic GABA_A_R-α5 anchoring.

### Rdx activation regulates lateral diffusion of GABA_A_R-α5

**Figure 5:**
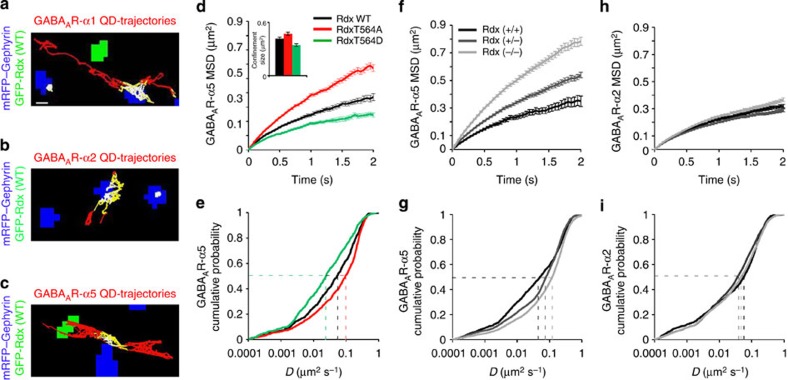
Rdx inactivation or depletion increases lateral diffusion of GABA_A_R-α5 within the plasma membrane. (**a**–**c**) Representative quantum dot (QD)-trajectories of GABA_A_R-α1, GABA_A_R-α2 and GABA_A_R-α5 in hippocampal neurons expressing mRFP–gephyrin (synaptic marker, blue) and GFP–Rdx (extrasynaptic marker, green). Receptor trajectories: outside of gephyrin clusters (red), peripheral (yellow) and inside clusters (white). (**d**,**e**) Diffusion of GABA_A_R-α5 outside of Rdx clusters in hippocampal neurons expressing Rdx-WT (black), RdxT564A (red) or RdxT564D (green). (**d**) Mean square displacement (MSD) versus time plot for GABA_A_R-α5-QD trajectories. Inset: average size of GABA_A_R-α5-QD confinement domains for Rdx-WT (0.416±0.017 μm^2^), RdxT564A (0.472±0.018 μm^2^) or RdxT564D (0.344±0.015 μm^2^) (RdxT564A versus Rdx-WT: *P*<0.05; RdxT564D versus Rdx-WT: *P*<0.01, *t*-test). (**e**) Cumulative probability plot for GABA_A_R-α5-QD diffusion coefficients. Dashed lines: median diffusion coefficients for RdxT564A (0.095 μm^2^ s^−1^: *P*<0.001) and RdxT564D (0.024 μm^2^ s^−1^: *P*<0.001) compared with Rdx-WT (0.053 μm^2^ s^−1^) (KS test). (**f**–**i**) Diffusion of GABA_A_R-α5 and GABA_A_R-α2 in hippocampal neurons derived from WT (+/+) (black), *Rdx* (+/−) (dark-grey) or *Rdx* (−/−) (light-grey) mice. (**f**) MSD versus time plot for GABA_A_R-α5-QD trajectories. Average size of GABA_A_R-α5-QD confinement size for *Rdx* (−/−) compared with *Rdx* (+/−) and WT (+/+);*P*<0.001 (*t*-test). (**g**) Cumulative probability plot of GABA_A_R-α5-QD diffusion coefficients. Dashed lines: median diffusion coefficients for *Rdx* (−/−) (0.121 μm^2^ s^−1^) compared with *Rdx* (+/−) (0.077 μm^2^ s^−1^) and WT (+/+) (0.050 μm^2^ s^−1^) (*P*<0.01, KS test). (**h**) MSD versus time plot for GABA_A_R-α2-QD trajectories. (**i**) Cumulative probability plot for GABA_A_R-α2-QD diffusion coefficients. Dashed lines: median diffusion coefficients for *Rdx* (−/−) (0.046 μm^2^ s^−1^) compared with *Rdx* (+/−) (0.039 μm^2^ s^−1^) and WT (+/+) (0.057 μm^2^ s^−1^) (*P*=NS, KS test). Scale bars, 500 nm. Error bars, mean±s.e.m. NS, not significant.

To address whether the alternation between surface membrane diffusion and Rdx-mediated confinement might be the underlying mechanism for the re-distribution of GABA_A_R-α5 molecules within the plasma membrane, we performed SPT analysis. QD-based SPT in the presence of either the unphosphorylatable (RdxT564A) or the phospho-mimetic mutant (RdxT564D) supported this view. Overall, GABA_A_R-α1 and GABA_A_R-α2 displayed lateral diffusion with a relatively high confinement at gephyrin clusters (depicted in blue; Fig. 5a,b). In contrast, GABA_A_R-α5 showed alternate diffusion and confinement at extrasynaptic Rdx clusters (depicted in green) and occasionally co-localized with synaptic gephyrin puncta (Fig. 5c). Overexpression of RdxT564A, which is unable to bind GABA_A_R-α5, induced a significant increase in the rate of diffusion of extrasynaptic GABA_A_R-α5 outside of Rdx clusters, compared with overexpression of Rdx-WT (Fig. 5d,e). An opposite effect was seen with RdxT564D, which caused lower GABA_A_R-α5 diffusion rates than Rdx-WT (Fig. 5d,e). Analysis of GABA_A_R-α5 SPT in neurons derived from either WT (+/+), heterozygous (+/−) or homozygous (−/−) *Rdx* KO mice yielded similar results. GABA_A_R-α5 showed significantly higher diffusion rates in *Rdx* (−/−) compared with *Rdx* (+/+) (Fig. 5f,g). Consistent with our hypothesis, *Rdx* (+/−), expressing about 50% of Rdx protein levels, displayed an intermediate GABA_A_R-α5 diffusion rate indicative of a dose-dependent relationship between GABA_A_R-α5 clustering and surface membrane diffusion. Such differences were not seen in the diffusion rates of either GABA_A_R-α2 (Fig. 5h,i) or GABA_A_R-α1 (Supplementary Fig. 5a and 5b) across the respective genotypic groups. Together, these findings indicate that the ability of Rdx to anchor GABA_A_R-α5 extrasynaptically determines whether or not the receptor is free to diffuse across the neuronal cell surface and eventually enters synaptic sites.

### Rdx dephosphorylation increases slowly decaying mIPSC events

**Figure 6:**
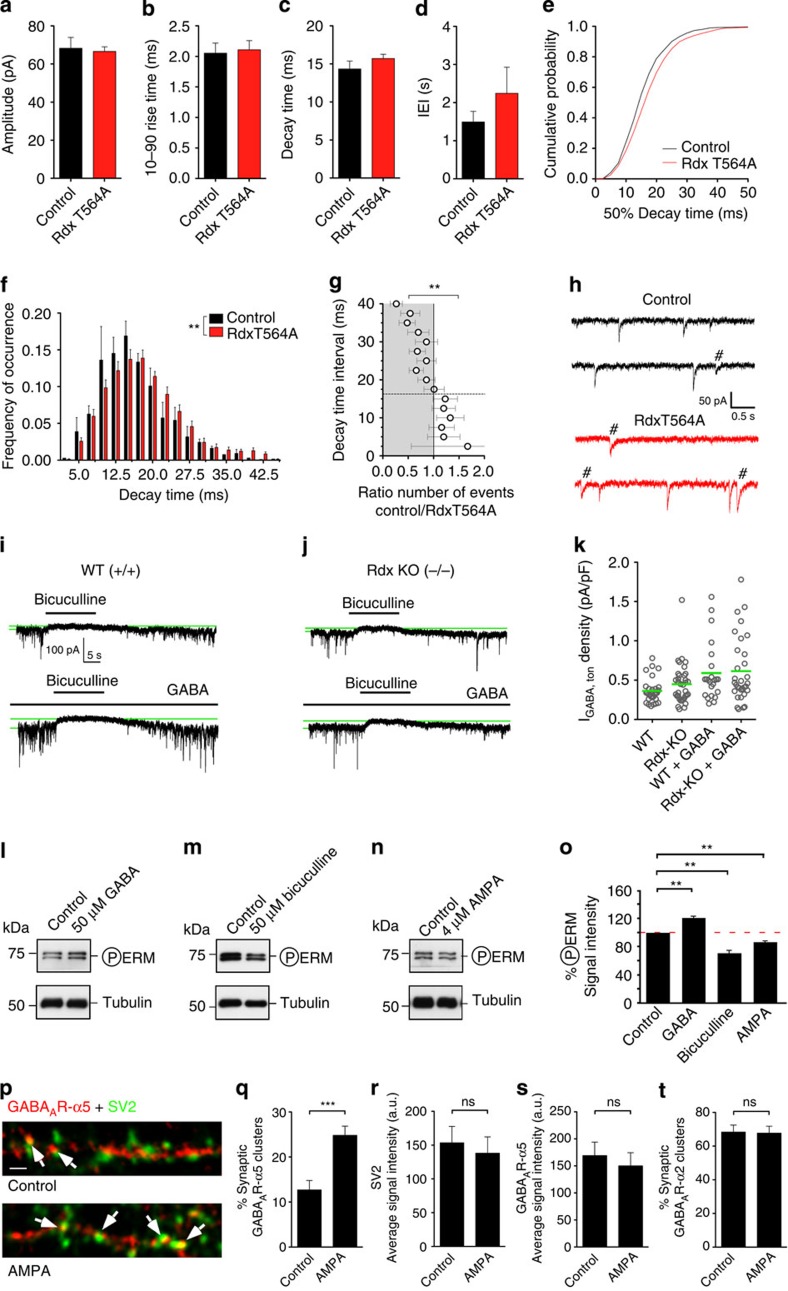
Rdx dephosphorylation depends on neuronal activity changes and increases the occurrence of slowly decaying mIPSCs. (**a**–**h**) Impact of RdxT564A or control vector expression on mIPSCs in hippocampal neurons. Mean±s.e.m. of (**a**) mIPSC amplitudes, (**b**) 10–90 rise times, (**c**) 50% decay times and (**d**) inter-event intervals (IEI) for RdxT564A versus control (*P*=NS, *t*-test). (**e**) Cumulative probability curves of mIPSC decay times for RdxT564A versus control. (**f**) Frequency distribution histogram of the data shown in **e** (**P<0.01; negative binominal regression; *n*=3; 12–13 cells per experiment). (**g**) Forrest plot of the data shown in **f**, representing ratios of the number of events for the decay times indicated. (**h**) Representative traces of GABAergic mIPSCs. ‘#' indicates slow events, typical for GABA_A_R-α5-mediated mIPSCs. (**i**–**k**) Tonic GABAergic currents in hippocampal neurons derived from (**i**) Rdx-WT (+/+) or (**j**) *Rdx*-KO (−/−) in the presence of 10 μM DNQX, 50 μM AP-5 and 500 nM tetrodotoxin. Currents were measured in the absence (upper panels) or presence of 100 nM GABA (lower panels). Application of 100 μM bicuculline blocked GABA currents. The amplitude of the bicuculline-sensitive tonic currents (distance between green lines in each panel) was converted into current density and plotted in a (**k**) dot plot (mean±s.e.m (pA per pF) for WT: 0.36±0.03, *Rdx*-KO: 0.45±0.05, *P*=0.12, *n*=28–35; WT+GABA: 0.59±0.08, *Rdx*-KO+GABA: 0.61±0.07, *P*=0.8, *n*=22–29, Mann–Whitney *U*-test). (**l**–**s**) Neuronal activity-dependent Rdx phosphorylation and synaptic re-distribution of GABA_A_R-α5. (**l**–**n**) Representative western blots detecting the phosphorylation of ERM proteins in hippocampal neurons on treatment with (**l**) 50 μM GABA, (**m**) 50 μM bicuculline or (**n**) 4 μM AMPA. (**o**) Quantification of ERM phosphorylation levels shown in **l**–**n** (control: set to 100%, GABA: 121.00±2.48%, bicuculline: 73.44±4.47%, AMPA: 86.00±1.15%; *n*=3–8; ANOVA and one-sample *t*-test against 100%). (**p**) Synaptic localization of GABA_A_R-α5 in neuronal dendrites (arrows) on excitatory synaptic potentiation (4 μM AMPA, 1 h). (**q**) % Synaptic GABA_A_R-α5 (control: 12.72±2.07%, AMPA: 24.82±2.04%). (**r**) SV2 signal intensities (control: 153.36±24.36 a.u., AMPA: 138.18±23.98 a.u.). (**s**) GABA_A_R-α5 signal intensities (control: 169.26±24.68 a.u., AMPA: 150.14±24.30 a.u.). (**t**) % Synaptic GABA_A_R-α2 remained unchanged (control: 68.39±4.12%, AMPA: 67.72±4.12%; *n*=3; 10–11 cells per experiment; ANOVA). Error bars, mean±s.e.m.; **P<0.01, ***P<0.001. NS, not significant.

GABA_A_R-α5 receptors regulate learning and memory^16^. Besides their role in tonic inhibition^19^, minor GABA_A_R-α5 populations contribute to hippocampal phasic inhibition^17^,^18^. To examine the physiological impact of GABA_A_R-α5 re-distribution through Rdx inactivation or depletion, we used whole-cell recordings of miniature inhibitory post-synaptic currents (mIPSCs) in hippocampal pyramidal neurons overexpressing RdxT564A. Average amplitude, 10–90% rise time, 50% decay time and inter-event intervals (IEI) were comparable to control neurons (Fig. 6a–d). Importantly, this manipulation significantly increased the frequency of occurrence of slowly decaying mIPSCs (Fig. 6e–h), a characteristic of GABA_A_R-α5-mediated events^18^. Comparable analyses of pyramidal neurons from acute hippocampal slices derived from *Rdx* −/− mice produced a similar shift towards more frequent occurrences of slowly decaying mIPSCs (Supplementary Fig. 6a,b). On the other hand, analysis of tonic GABAergic currents in the absence or presence of 100 nM GABA, revealed no differences between the genotypes (Fig. 6i–k). Analogous experiments on WT neurons using Rho kinase II inhibitor, which increased synaptic GABA_A_R-α5 levels (see Fig. 3d,e), also revealed no effect on tonic GABAergic current densities (mean±s.e.m (pA per pF) for control: 0.248±0.071, RhoKII-I: 0.307±0.070, *P*=0.554; control+GABA: 0.484±0.174; RhoKII-I+GABA: 0.701±0.213, *P*=0.435, Mann–Whitney *U*-test, *n*=11–14). These results are consistent with a computational simulation (Supplementary Fig. 6c,d). It implies that the observed reduction in the number of extrasynaptic receptors would only give a marginal shift in tonic current, which is in keeping with our electrophysiological observations. Thus, higher GABA_A_R-α5 concentrations at synapses (Figs 2a,b and 4a–d) modulate inhibitory phasic transmission without detectable effects on tonic inhibition.

### Rdx phosphorylation depends on neuronal activity changes

To validate the physiological relevance of these observations, we altered GABAergic and glutamatergic transmission upstream of RhoA signalling^30^. GABA application increased, whereas application of AMPA or the GABA_A_R blocker bicuculline decreased Rdx phosphorylation levels (Fig. 6l–o). In support of our earlier finding, demonstrating that decreased Rdx phosphorylation induces higher synaptic GABA_A_R-α5 levels (Fig. 2), activation of AMPA receptors evoked GABA_A_R-α5 enrichment at synaptic sites (Fig. 6p,q). The AMPA induced increase in synaptic GABA_A_R-α5 did not stem from changes in either synapse number or GABA_A_R-α5 expression levels (Fig. 6r,s) and did not alter the synaptic concentration of GABA_A_R-α2 (Fig. 6t and Supplementary Fig. 6e,f). Hence, the observed increase in synaptic GABA_A_R-α5 is likely a consequence of receptor re-distribution following upstream activity changes.

### Radixin depletion in mice impairs short-term memory

We next sought to address the physiological relevance of depleting the extrasynaptic GABA_A_R-α5 scaffold protein Rdx *in vivo*. Initial behavioural tests conducted on *Rdx* (−/−) mice revealed no deficits in motor learning, muscle strength and spontaneous alternation behaviour (Supplementary Fig. 7a–c). Likewise, no differences in anxiety-related behaviour emerged between *Rdx* (−/−) and WT (+/+) mice (Supplementary Fig. 7d,e).

**Figure 7:**
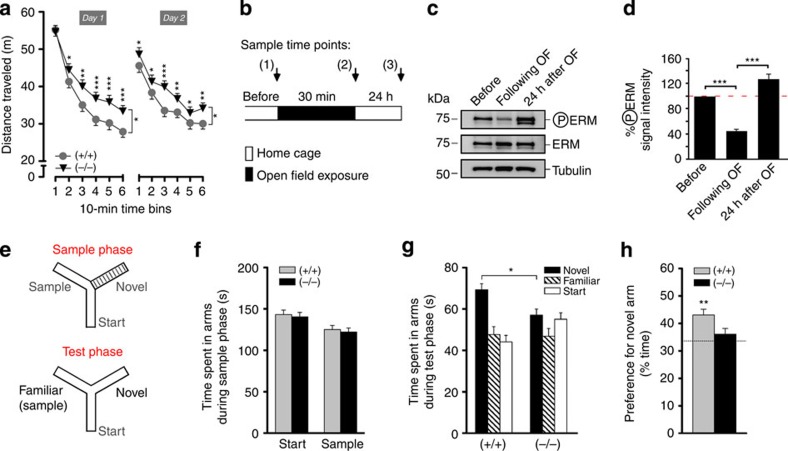
Rdx depletion impairs hippocampal-dependent short-term memory. (**a**) Locomotor exploratory activity in the open field (OF). (repeated measures (RM) ANOVA: bins × genotype, *F*_5,275_=2.64, *P*<0.05; genotype, *F*_1,55_=4.30, *P*<0.05). WT (+/+)=29, *Rdx* (−/−)=28. (**b**–**d**) Open field experiment in C57Bl6-WT mice and analysis of phosphorylated-ERM (PERM) protein levels at three time points: (1) before, (2) following and (3) 24 h after OF testing. (**c**) Representative PERM levels in hippocampal neurons derived from the animals tested. (**d**) Quantification reveals decreased PERM levels following OF exposure (44.43±3.75%), compared with controls (before: set to 100%) and increased PERM levels after 24 h (126.59±5.29%); (ANOVA: PERM level × time point: *F*_2,9_=91.061, *P*<0.0001, *n*=3–6). (**e**) Y-maze spatial recognition test. In the sample phase, mice were allowed to freely explore the start and a sample arm for 5 min. Access to the other sample arm, which later served as the novel arm, was blocked in this phase. Following a delay interval, the mice were tested for novelty preference by returning them to the maze with free access to all three arms for 3 min. (**f**) Mean time spent in each arm during the sample phase revealed no difference between WT (+/+) (*n*=8) and *Rdx* (−/−) (*n*=8) mice (ANOVA; arms × genotype: *F*_1,14_=0.00, *P*>0.90). (**g**) Mean time spent in all arms during the novelty test phase. WT (+/+) mice spent significantly more time in the novel arm (black bar), compared with *Rdx* (−/−) mice (genotype: *F*_1,14_=8.79, *P*<0.05). (**h**) Preference for the novel arm against chance level performance (33%). One-sample *t*-test: (WT (+/+) mice, *t*_7_=5.00, *P*<0.005; *Rdx* (−/−) mice, *t*_7_=1.27, *P*>0.1. Error bars, mean±s.e.m., *P<0.05, **P<0.01, ***P<0.001.

Testing for locomotor exploration revealed a clear difference in the activity profile between the two genotypes. Here *Rdx* (−/−) mice exhibited a significant within-session decrease in the rate of habituation to the open field (Fig. 7a). We therefore examined whether testing in the open field was sufficient to alter phosphorylation levels of the critical T564 residue in the Rdx molecule in WT mice. A 30 min exposure to the open field in WT mice significantly reduced Rdx phosphorylation levels (Fig. 7b–d), which has been shown to cause re-distribution of GABA_A_R-α5 into synapses in different cellular assays (see Figs 2f,i, 3a,e and 4i,j and Fig. 6n–q). This effect recovered in a separate group of WT mice that was analysed 24 h after OF exposure (Fig. 7b–d), indicating that Rdx phosphorylation represents a dynamic process that is highly sensitive to *in vivo* testing, and therefore relevant for behavioural function.

Previous studies have shown that genetic depletion of GABA_A_R-α5 or administration of a specific GABA_A_R-α5 inverse agonist (α5IA) enhances cognitive performance^16^,^31^. In line with this, we first examined for short-term spatial recognition memory in the Y-maze task (Fig. 7e), whereby mice typically display an intrinsic preference to explore novel over familiar arms. No differences between WT (+/+) and *Rdx* (−/−) were observed during the sample phase (Fig. 7f). Hence, any impairment in the subsequent test phase cannot be attributed to inadequate familiarization. As illustrated (Fig. 7g,h), *Rdx* (−/−) mutants were grossly impaired in the subsequent test phase. They failed to show any preference for the novel arm above chance performance, indicating their inability to discriminate between novel and familiar arms. This severe deficit in spatial familiarity judgment may also contribute to the short-term habituation deficit seen in the open field (Fig. 7a), and could represent a more general short-term memory deficiency. Despite this, administration of the GABA_A_R-α5 receptor inverse agonist (α5IA) prior to the Y-maze test did not enhance performance in either the WT (+/+) or *Rdx* (−/−) mice (Supplementary Fig. 7f–h).

### Radixin depletion in mice impairs reversal learning

**Figure 8:**
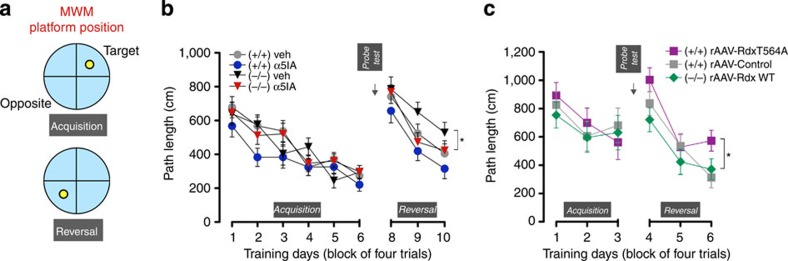
Rdx depletion impairs reversal learning. (**a**) Morris water maze (MWM) assessment of spatial reference memory and reversal learning. Yellow circle: platform position. (**b**) MWM with α5IA administration. Acquisition: all treatment groups successfully acquired the task with performance improving progressively across training days (RM ANOVA: main effect of days *F*_5,265_=28.92, *P*<0.0001). Reversal learning: a reversal effect (a severe drop of performance from the last acquisition day to the first reversal day) emerged in all treatment groups (RM ANOVA: effect of days *F*_1,53_=125.60, *P*<0.0001). *Rdx* (−/−) vehicle-treated mutants were significantly impaired in the course of new learning (RM ANOVA: days: *F*_2,106_=35.24, *P*<0.0001; genotype: *F*_1,53_=4.84, *P*<0.05; treatment: *F*_1,53_=5.04, *P*<0.05). *Post-hoc* restricted analyses show that *Rdx* (−/−) α5IA-treated mutants performed better than *Rdx* (−/−) veh-treated mice (*F*_1,26_=3.873, *P*=0.06). (*Rdx* (−/−) α5IA versus WT (+/+) veh: *F*_1,28_=0.001, *P*=0.975). Data were based on 57 mice (WT (+/+) veh=15; WT (+/+) α5IA=14; *Rdx* (−/−) veh=13; *Rdx* (−/−) α5IA=15). (**c**) MWM with bilateral hippocampal rAAV-Rdx expression. Acquisition: all virus-treated groups performed successfully across training days (RM ANOVA: main effect of days *F*_2,36_=5.23, *P*<0.05). Reversal learning: a reversal effect appeared in all virus-treated groups (RM ANOVA: effect of day *F*_1,18_=5.35, *P*<0.05). Notably, *Rdx* (−/−) rAAV-Rdx-WT-treated mutants performed significantly better than *Rdx* (+/+) rAAV-Rdx-T564A-treated control mice (*P*<0.05) and did not differ from *Rdx* (+/+) rAAV-control-treated mice (*P*=0.501) in reversal learning as shown by *post-hoc* pair wise comparison. (RM ANOVA: days: *F*_2,36_=31.36, *P*<0.0001; virus-treated groups: *F*_1,18_=3.02, *P*=0.074). Data were based on 21 mice (*Rdx* (+/+) rAAV-Rdx-T564A=7; *Rdx* (−/−) rAAV-Rdx-WT=7; *Rdx* (+/+) rAAV-control=7). Error bars, mean±s.e.m., *P<0.05.

Given that the cognitive enhancing effect of α5IA has been reported in tasks that require greater cognitive demand and/or flexibility^31^,^33^, we therefore examined four treatment groups (WT (+/+) veh; WT (+/+) α5IA; Rdx (−/−) veh; Rdx (−/−) α5IA) for spatial reference memory and reversal learning in the Morris water maze task^32^ (Fig. 8a). In the spatial reference memory (RM) acquisition, all treatment groups successfully acquired the task and showed a clear memory for the platforḿs location in the probe test (Fig. 8b and Supplementary Fig. 7i). Average swim speeds were comparable for all four treatment groups (analysis of variance (ANOVA): genotype *F*_1,53_=1.968, *P*>0.1). On the first day of reversal learning, a reversal effect was evident in all treatment groups, although *Rdx* (−/−) veh-treated mice were significantly slower in adapting their search behaviour to the changed location of the escape platform (Fig. 8b). On the other hand, *Rdx* (−/−) α5IA-treated group performed comparably better than *Rdx* (−/−) veh-treated mice, indicating a crucial role for GABA_A_R-α5 in the reversal learning deficits seen in *Rdx* (−/−) veh-treated mice.

We were keen to examine further the specific role of hippocampal (the predominant region of GABA_A_R-α5 expression) Rdx phosphorylation in the reversal deficit seen in *Rdx* (−/−) mice. Therefore, we re-expressed different Rdx-mutant constructs using bilateral hippocampal stereotaxic recombinant adeno-associated virus (rAAV) gene delivery. *Rdx* (−/−) and WT (+/+) mice expressing the different constructs were examined for reversal learning (Fig. 8c and Supplementary Fig. 7j-k) as described above. Here *Rdx* (−/−) mice expressing rAAV-Rdx-WT showed comparable performance to WT (+/+) rAAV-control mice in the course of reversal learning, suggesting that hippocampal Rdx re-expression is sufficient to normalize performance. Moreover, hippocampal expression of the GABA_A_R-α5-binding deficient Rdx mutant (rAAV-Rdx-T564A; see Supplementary Fig. 7j and Fig. 2e) in WT (+/+) mice induced a specific reversal learning deficit (Fig. 8c) resembling that seen in *Rdx*-KO (−/−) veh mice (Fig. 8b). This reinforces our suggestion, that Rdx phosphorylation, which is critical for GABA_A_R-α5-Rdx uncoupling, underlies the appearance of the reversal learning phenotype in the *Rdx* (−/−) mice.

Altogether we show that Rdx phosphorylation is a dynamic process that is relevant for behavioural function and that loss of Rdx phosphorylation interferes with behavioural measures involving cognitive function.

## Discussion

**Figure 9:**
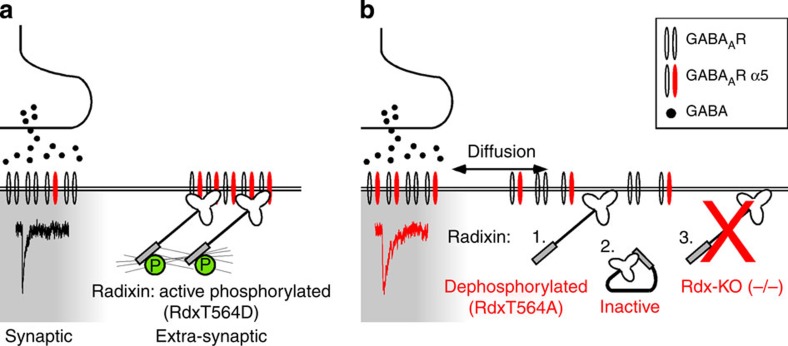
Model: radixin-dependent regulation of synaptic and extrasynaptic GABA_A_R-α5 exchange. (**a**) Active phosphorylated Rdx (mimicked by RdxT564D expression) binds GABA_A_R-α5 and traps receptors extrasynaptically via its C-terminal interaction with the F-actin cytoskeleton (thin black lines). (**b**) (1) Rdx dephosphorylation (mimicked by RdxT564A expression), (2) inactive cytosolic Rdx or (3) genetic depletion of Rdx (*Rdx*-KO (−/−)) releases surface membrane GABA_A_ receptors containing the α5 subunit from its extrasynaptic anchor, leading to lateral surface membrane diffusion and synapse entry, thereby modulating inhibitory synaptic transmission. Together, our data propose the concept that extrasynaptic GABA_A_R-α5 clusters represent potential plasma membrane reservoirs in the regulation of synaptic plasticity.

In the present study, we identified a neuronal activity-regulated RhoA–ROCK–Rdx pathway that uncouples GABA_A_R-α5 from extrasynaptic Rdx surface membrane clusters in a phosphorylation-dependent manner. The release of GABA_A_R-α5 from Rdx-mediated anchorage to the actin cytoskeleton enhances lateral receptor diffusion, alters synaptic receptor concentration and induces a shift towards more frequent occurrences of slowly decaying GABA_A_R-α5-specific mIPSCs (Fig. 9). In addition to their relevance in tonic inhibition, our study has identified a role for GABA_A_R-α5-containing receptors in phasic synaptic transmission. This is critically dependent on Rdx, which regulates extrasynaptic release and trapping of GABA_A_R-α5 receptors. *In vivo*, Rdx depletion consistently increases the accumulation of GABA_A_R-α5 receptors at synapses and impairs specific aspects of learning and memory. The deficits can be normalized by systemic GABA_A_R-α5 inhibition or hippocampal re-expression of Rdx-WT in *Rdx* KO mice. Behavioural testing of WT mice induces changes in Rdx phosphorylation, consistent with our suggestion that the Rdx T564 phosphorylation switch, which regulates the concentrations of GABA_A_R-α5 at synapses, participates in behavioural processes.

Receptor storage pools have been described at intracellular sites and are known to exchange receptors via endocytic recycling. Under LTP conditions, local submembrane receptor storage is required for the efficient supply of molecules on a fast time scale^4^. Further evidence has been provided recently by a study showing that the minimum requirement for LTP is a reserve pool of non-synaptic AMPA receptors, whether at the cell surface or at intracellular recycling endosomes^34^.

The present study extends this view by proposing that extrasynaptic plasma membrane receptor clusters, regulated through an extrasynaptic scaffold protein, could also constitute reservoirs to rapidly supply receptors in the modulation of synaptic function. Although different neurotransmitter receptors exist at extrasynaptic sites, GABA_A_R-α5 receptors are unique in their ability to accumulate in distinct clusters beyond inhibitory synaptic contacts^7^,^22^,^35^,^36^. Therefore, reversible extrasynaptic receptor clustering might be well suited to control synaptic plasticity, and support the formation of new memory, as well as the suppression of old (and ill-adaptive) memory.

Evidence that synaptic and extrasynaptic receptors play different physiological roles comes from NMDA receptors (NMDARs), which are also known to localize at both sites. NMDARs mediate opposing effects and activate different intracellular signalling pathways, depending on whether or not they are in apposition to glutamatergic terminals. Imbalances between synaptic and extrasynaptic NMDARs contribute to neuronal dysfunction^37^. Furthermore, the balance between excitatory and inhibitory receptors contributes to neuronal homeostasis and NMDAR activation influences GABA_A_R lateral mobility and GABA_A_R clustering at inhibitory synapses^38^,^39^. Accordingly, our observation that AMPA application doubles the synaptic localization of GABA_A_R-α5 receptors (Fig. 6n–q), suggests that Rdx uncoupling might also contribute to homeostatic regulation in the hippocampus.

Yet, the functional significance of GABA_A_R-α5 clustering at extrasynaptic sites beyond axo-dendritic contacts has remained unclear. GABA_A_R-α5 receptors are highly sensitive to GABA and desensitize slowly, two optimal features in regulating tonic inhibition^19^. Other studies have identified GABA_A_R-α5-containing receptors at post-synaptic sites^21^, albeit to a lesser extent (10–25% synaptic versus 75–90% extrasynaptic receptors^7^,^22^), compared with GABA_A_R-α1- and GABA_A_R-α2- containing receptors (33–50% synaptic versus 50–67% extrasynaptic receptors^40^). Accordingly, pharmacological studies have revealed a contribution of GABA_A_R-α5 in mediating slow synaptic inhibition in the hippocampus^17^,^18^, indicating that GABA_A_R-α5 receptors mediate differential effects in tonic and phasic inhibition. Here the SPT data (Fig. 5d–g) and the immunocytochemical analysis of GABA_A_R-α5 cluster size (Fig. 4f,g) extend this view by showing that synaptic receptor clusters can be increased at the expense of extrasynaptic neurotransmitter receptors. Lateral cell surface diffusion *per se* is, however, a random and non-directional process that requires differential confinement through affinity trapping to concentrate receptors^5^.

Our data suggest that Rdx acts as an affinity trap to concentrate GABA_A_R-α5 at extrasynaptic sites in a phosphorylation-dependent manner. Upon release from Rdx binding, we show that GABA_A_R-α5 receptors freely diffuse laterally across the plasma membrane and eventually integrate into synaptic contacts. Overexpression of unphosphorylated Rdx or Rdx depletion increases synaptic GABA_A_R-α5 concentration (Figs 2 and 4). This scenario requires a second receptor affinity trap at the GABAergic post-synaptic site to confine receptors in apposition to GABA-releasing terminals. The post-synaptic GABA_A_R clustering protein gephyrin is a prominent candidate in this respect, as it directly binds to, and anchors various GABA_A_R subunits (α1, α2, α3, β2 and β3), one of which (β3) assembles with the α5 and γ2 subunit into functional receptors^6^,^9^,^10^,^11^,^21^. Pentameric assemblies of GABA_A_R subunits^41^ could therefore possess multiple binding sites for differential scaffold interactions at synapses and at extrasynaptic sites. This might also reflect the different diffusion properties of individual GABA_A_R subunits, observed in our study. Previous studies have demonstrated that adjusting the affinity between GABA_A_R subunits and gephyrin modulates receptor accumulation at synapses^42^. Our data extends this view by suggesting an additional mechanism, whereby the amount of sequestered surface GABA_A_Rs in the extrasynaptic membrane participates in determining the equilibrium of receptor number at synapses.

Interestingly, re-distribution of GABA_A_R-α5 into synapses alters mIPSCs but does not lead to detectable changes in tonic inhibition. Several possibilities may contribute to this observation: (i) The majority of GABA_A_ receptors are located at extrasynaptic sites^22^,^40^, (ii) Although GABA_A_R-α5 mediates about 70% of the of the tonic conductance in hippocampal pyramidal cells, 30% depends on GABA_A_R-δ^43^, (iii) The open probability of extrasynaptic GABA_A_Rs is in the range of about 10% (refs 44, 45). Therefore, in agreement with our experimental data (Fig. 6i–k) and the computational simulation of inhibitory tonic currents (Supplementary Fig. 6c,d), a shift of GABA_A_R-α5 receptors into synapses at the expense of extrasynaptic receptors is not expected to cause major alteration in tonic currents. We therefore propose that Rdx-mediated GABA_A_R-α5 re-distribution constitutes a mechanism to primarily modulate phasic inhibition.

Depletion of the GABA_A_R-α5 gene had been shown to improve spatial learning in mice^16^. At the molecular level, *Rdx*-KO mice bear an opposite characteristic by showing an increased proportion of synaptic GABA_A_R-α5 receptors. Here a parsimonious account of the Y-maze deficit in familiarity judgment and habituation deficit in the open field exhibited by *Rdx* (−/−) mice may point to an impairment in short-term memory^46^. On the other hand, an inability to suppress old memory that interferes with the establishment of new learning may account for the reversal learning deficit of the *Rdx* (−/−) mice. This is in apparent contrast to the phenotypes of *GABA*_*A*_*R*-α*5* KO mice that may indicate enhanced cognitive flexibility. Hence, it is reasonable to attribute the cognitive phenotype in *Rdx* (−/−) mice to an increase in synaptic GABA_A_R-α5 receptors rather than the reduction in extrasynaptic receptors, which is common to both the *Rdx* (−/−) and *GABA*_*A*_*R*-α*5* KO mouse models.

The specific inhibition of GABA_A_R-α5 by an inverse agonist (α5IA) in *Rdx* (−/−) mice improves their performance in reversal learning, thereby indicating a significant contribution of GABA_A_R-α5 in the phenotype seen in *Rdx* (−/−) mice. In addition hippocampal re-expression of WT Rdx in the KO background normalizes the reversal learning deficit, while expression of GABA_A_R-α5 binding deficient mutant form of Rdx in WT mice recapitulates the deficit seen in *Rdx* (−/−) mice. Thus, hippocampal Rdx phosphorylation and associated changes in GABA_A_R-α5 re-distribution seem to play a crucial role in the reversal deficit seen in *Rdx* (−/−) mice.

Given that Rdx is also involved in other cellular processes^14^, we cannot rule out a possible involvement of alternative Rdx interacting partners in some of the behavioural phenotypes seen in *Rdx* (−/−) mice. However, our findings reveal the functional significance of a dynamic regulation of Rdx and GABA_A_R-α5 localization to behavioural and cognitive function. This is consistent with the observation that phosphorylation of RdxT564 is highly responsive to general behavioural manipulations.

Together, our model proposes that extrasynaptic GABA_A_R-α5 receptor clusters constitute plasma membrane reservoirs for the rapid supply of receptors into GABAergic synapses. It provides a starting point to address: (i) whether the synaptic–extrasynaptic exchange of other neurotransmitter receptors is regulated by similar mechanisms and (ii) the extent to which plasma membrane receptor reservoirs contribute to the regulation of synaptic plasticity or neuronal function. Rdx is currently the only known scaffold protein to cluster inhibitory neurotransmitter receptors extrasynaptically. Competition between synaptic gephyrin and extrasynaptic Rdx for receptor binding might ultimately determine the synaptic versus extrasynaptic equilibrium of GABA_A_ receptors.

The GABA_A_R-α5 gene locus has been associated with schizophrenia and autism spectrum disorders. Similarly, GABA_A_Rs are involved in several pathological conditions^13^,^47^. Understanding Rho–ROCK–Rdx signalling and its implication in synaptic/extrasynaptic GABA_A_ receptor ratios may therefore help to provide novel insights into multiple neuronal disease processes.

## Methods

### Constructs

RdxT564D and RdxT564A were generated using a site-directed mutagenesis kit (Stratagene, LaJolla, CA) and cloned as XhoI—KpnI fragments into pEGFP-N2 vector (BD Bioscience, Heidelberg, Germany) or as KpnI–SacI fragments into pBK-CMV-myc (Stratagene). To integrate RdxT564A–green fluorescent protein (RdxT564A–GFP) in the pdsAAV-TNNT2 transfer plasmid, the DNA was amplified from the previously generated pEGFP-N2-RdxT564A construct using primer pairs containing SalI–XhoI sites (5′- ATCCTCTAGAGTCGACATGCCGAAGCCAATCAATGTAAGAG -3′; 5′- CCGTAGATCTCTCGAGTTACTTGTACATCTCGTCCATGC -3′). Rdx-WT–GFP–pAAV was generated by subcloning Rdx-WT-pEGFP-N2 as HindIII–KpnI fragment into the previously generated RdxT564A–GFP–pAAV vector. The following constructs have been previously described: pGEX-5X-1-GABA_A_R-α5 TMIII-TMIV intracellular loop^7^, pGEX-5X-1-GABA_A_R-α2 TMIII-TMIV intracellular loop^3^, pEXV-RhoN19, pEXV-RhoV14 and pEXV-RacV12 (ref. 25). All constructs were verified by dideoxy sequencing.

### Antibodies

The following antibodies were used for western blotting (WB): rabbit anti-ERM (1:1,000; Cell Signaling, Danvers, MA); rabbit anti-PERM (1:2,000; Cell Signaling); mouse anti-α-tubulin (1:15,000; Sigma, Taufkirchen, Germany); rabbit anti-myc (1:4,000; Sigma); mouse anti-PSD-95 (1:2;000; ABR, Golden, CO); mouse anti-gephyrin (BD Bioscience, CA); rat anti-Rdx (R21, 1:50; ref. 7^7^); rabbit anti-GABA_A_ receptor α5 (1:1,000, Acris, Herford, Germany); rabbit anti-GABA_A_ receptor α1 (1:1,000, Serotec, Oxford, UK); mouse anti-β-actin (1:3,500; Sigma); mouse anti-SNAP25 (1:2,000; BD Bioscience); rabbit anti-syntaxin1a (1:1,000; GeneTex, Irvine, CA); peroxidase-conjugated goat anti-rat, goat anti-rabbit and goat anti-mouse (all 1:15,000; Dianova, Hamburg, Germany).

The following antibodies were used for immunofluorescence: rabbit anti-GABA_A_ receptor α5 (1:50); rabbit anti-GABA_A_ receptor α2 (1:250; Synaptic Systems, Göttingen, Germany); rabbit anti-VGAT (1:500; Synaptic Systems); mouse anti-synaptic vesicle (SV2, 1:100; Hybridoma Bank, University of Iowa, IA); mouse anti-gephyrin (mab7a, 1:250; Synaptic Systems); rat anti-Rdx (R21, 1:50; ref. 7); mouse anti-GM130 (clone 35, 1:500; BD Bioscience); CY3-conjugated donkey anti-rabbit, CY3-conjugated donkey anti-rat and CY5-conjugated donkey anti-mouse (all 1:500; Dianova).

### Cell culture and transfection

Primary cultures of hippocampal neurons were prepared from *Rdx*-KO (−/−) or WT (+/+) littermate control mice at postnatal day 0, as described^3^,^7^. Cells cultured for 10 to 12 days *in vitro* (DIV) were used for transfection by a calcium phosphate co-precipitation protocol^3^. About 16 h after transfection cells were fixed and processed for immunocytochemistry.

### Glutathione *S*-transferase-(GST)-pulldown assay

Human embryonic kidney (HEK293 (ATCC, Manassas, VA)) cells were cultured in 3.5-cm or 10-cm cell culture dishes as described^3^. For pull down experiments, 24 h after transfection of RdxT564D-myc or RdxT564A-myc, HEK293 cells were harvested in 1 ml phosphate-buffered saline (PBS) containing 1% Triton X-100 (Merck, Darmstadt, Germany), 1 mM PMSF (Calbiochem, Darmstadt, Germany) and complete protease inhibitor (Roche, Mannheim, Germany). *E. coli* BL21 lysates were obtained 24 h after electroporation of pGEX-5X-1-GABA_A_R-α5 or -GABA_A_R-α2 TMIII-TMIV by sonification in 100 mM HEPES (pH 7.9) and 400 mM NaCl supplemented with 10% glycerol (v/v) (Roth, Karlsruhe, Germany) followed by centrifugation at 10,000*g* for 30 min. Precipitation was performed as described^3^.

### Immunocytochemistry

Primary hippocampal neurons (12–14 DIV) from *Rdx*-KO (−/−) and WT (+/+) littermate control mice were fixed in PBS containing 4% paraformaldehyde (PFA) (w/v) and 4% sucrose (w/v) (12 min) and processed further for immunofluorescence staining as described^3^. To label the nucleus, Hoechst 33342 (1:50,000; Thermo Scientific Pierce, Waltham, MA) was used in combination with the secondary antibody.

### Preparation of synaptosomes

Whole brains of adult *Rdx*-KO (−/−) and WT (+/+) littermate control mice were processed, as described^3^, and separated on a discontinous 1.2–0.85 M sucrose gradient for 2 h at 82,500*g* (4 °C). Synaptosomal fractions were recovered from the gradient at molarities of 1.0–1.2 M. After substitution of 0.5% Triton X-100 (v/v), fractions were incubated for 15 min at 4 °C and subsequently centrifuged at 70,000*g* for 1 h. Resulting supernatants, constituting the pre-synaptic fractions, were removed. Pellets constituting the PSD fractions and all other fractions were substituted with 0.5% TX-100 and incubated for 15 min at 4 °C. Protein concentrations were assayed and adjusted using a bicinchonic acid (BCA) assay (Pierce Biotechnology, Rockford, IL). Samples were boiled in SDS sample buffer and analysed by WB.

### Analysis of PERM protein levels

For RhoA GTPase expression, HEK293 cells were transfected with the corresponding constructs and cultured for 16 h. For drug treatments, DIV 14 mouse hippocampal neurons were incubated with 50 μM Rho-Kinase-II-Inhibitor (Calbiochem), 50 μM bicuculline (Sigma), 50 μM GABA (Sigma) or 4 μM AMPA (Sigma). The solvent DMSO (Sigma-Aldrich, Taufkirchen, Germany) was used as a control. The following steps were performed at 4 °C: cells were washed once in PBS and harvested in PERM-lysis buffer, containing 1% Triton X-100, 50 mM NaF (Applichem, Darmstadt, Germany), 2 mM Na_3_OV_4_ (Applichem), 2 mM EDTA (Applichem), 10 mM β-glycerol phosphate (Sigma) and 10 mM sodium pyrophosphate (Sigma) in PBS. After incubation for 30 min, lysates were centrifuged at 1,000*g* for 10 min. The resulting supernatants were boiled in SDS sample buffer after adjustment of protein concentrations using a BCA assay (Pierce Biotechnology). Samples were analysed by WB. For figure displays, blots were cropped. Full scans of uncropped blots are presented in Supplementary Fig. 8.

### Blockade of exocytosis in primary hippocampal neurons

To interfere with Golgi vesicle segregation and exocytosis, primary hippocampal neurons were treated with 500 nM brefeldin A (solvent: DMSO) and 500 nM N-ethylmaleimide (solvent: HEPES buffer pH 7.0) (Sigma) for 1 h at 37 °C. The solvent was used as control. Neurons were then washed twice in PBS and further processed for immunocytochemistry, as described.

### Image processing and statistical analysis of IF and WB data

For evaluation of relative WB signal intensities, films were scanned with a resolution of 600 dpi and signals subsequently analysed using the ImageJ analysis software (version 1.38; National Institutes of Health, NIH). Signal intensities of phosphorylated PERM protein levels or protein levels of synaptosomal preparations were normalized, against loading control signals. Furthermore, protein signal intensities after drug treatment or after Rdx depletion (*Rdx*-KO (−/−)), respectively, were compared with control treatments or to WT (+/+) signal intensities that were set to 100%. Statistical analysis was performed with Microsoft Excel. The student's *t*-test and ANOVA was used to assess statistical significance.

Fluorescence imaging was carried out with an upright Olympus FluoView TMFV1000 (Olympus, Hamburg, Germany) laser scanning confocal microscope using a × 63 objective. For simultaneous double-channel fluorescence, images were taken in a sequential scanning mode. Confocal images from multiple cells of both genotypes were obtained using identical photomultiplier settings throughout all experiments. Experiments were replicated at least three times using different culture preparations. Images were saved as overlay TIF files and further analysed using MetaMorph 7.1 (Universal Imaging, Downingtown, PA). Regions of interests from distal neurites or cell somata were defined throughout multiple image acquisition frames. pEGFP signals were used to outline neuronal morphology. Overlay TIF files were separated in green, red and blue channels using the ‘colour separate' function and regions of interests were transferred from the overlay to each channel. For definition of image thresholds, brightness was adjusted using the ‘inclusive thresholding state' function. Average fluorescent intensities were calculated from average grey values of the corresponding channel. Fluorescence intensity scans across the somata were performed using Metamorph́s ‘linescan' function. Fluorescent intensities were calculated from grey values at each pixel along the selected region and plotted as a function of positions along the line.

To analyse synaptic GABA_A_R-α5, -α2 or Rdx levels, separate signals in co-localization with the pre-synaptic marker protein SV2 (magenta signals) were determined using the ‘threshold image' function and quantified with the function ‘count cells'. Extrasynaptic GABA_A_R-α5 or Rdx levels were defined as non-colocalizing clusters with SV2 (red signals substracted by magenta signals). The percentage of synaptic clusters was calculated as follows: (yellow signals/red signals) × 100%. Within one experiment, threshold adjustments were unchanged between all conditions. For better visualization, signal intensities were presented in false colour (SV2, blue to green; pEGFP green to grey). Synaptic cluster sizes were assessed by Metamorph-based analysis using the ‘Integrated Morphometry Analysis' tool, calculating the area of single objects. Statistical analysis was performed with Microsoft Excel or SPSS 15.0 (SPSS Inc. Chicago, IL). Statistical significance was assessed with a two way ANOVA (genotype × receptor localization) or the Student's *t*-test. All values from quantitative data represent the mean±s.e.m. from *n* independent experiments.

### Quantum Dot (QD)-based single particle tracking (SPT)

Primary cultures of hippocampal neurons were prepared from E18 mRFP–gephyrin knock-in mice^48^. GABA_A_Rs were labelled with QDs as described^39^. Briefly, neurons were labelled and imaged in MEM recording medium consisting of MEM (Invitrogen, Paisely, UK) supplemented with 10 mM HEPES, 33 mM glucose, 2 mM glutamine, 1 mM sodium pyruvate and B27 at 37 °C. Cells were incubated for 5 min with primary antibodies, washed, incubated for 5 min with biotinylated secondary Fab and washed again. Cells were then incubated for 1 min with streptavidin-coated QDs emitting at 655 nm (1 nM, Invitrogen) in borate buffer (50 mM). Neurons were observed using an inverted microscope (IX71, Olympus) equipped with an oil-immersion objective (Olympus, × 60, NA 1.45), a xenon lamp, cooled CCD camera Cascade+128 (Roper Scientific, Tucson, Arizona) or ORCA II ER (Hamamatsu photonics, Hamamatsu, Japan). Fluorescent signals were detected using appropriate filter sets for QD (D455/70x and HQ655/20), and mRFP (D535/50 and E590lpv2). The movement of GABA_A_R QDs on the proximal dendrites was recorded with an integration time of 30 ms with 500 consecutive frames (15 s). The recording was done up to 30 min after labelling.

Tracking and analysis of QDs is described in detail elsewhere^49^. Briefly, QDs were detected by cross-correlating the image with a Gaussian model of the point spread function, and the diffusion parameters were calculated using custom software^9^ using Matlab (The Mathworks Inc., Natick, Massachusetts). Single QDs were identified by intermittent fluorescence (that is, blinking). The spots in a given frame were connected with the maximum likely trajectories estimated on previous frames of the image sequence. Only trajectories with at least 15 consecutive frames were used for further analysis. Synaptic areas were defined by processing mRFP–gephyrin images with the multidimensional image analysis interface. GABA_A_R QDs were classified as ‘synaptic' when the trajectories overlapped with synaptic areas. The trajectories were considered ‘extrasynaptic' when their distance to the synapse was ⩾2 pixels. The mean square displacement (MSD) was calculated using equation (1).


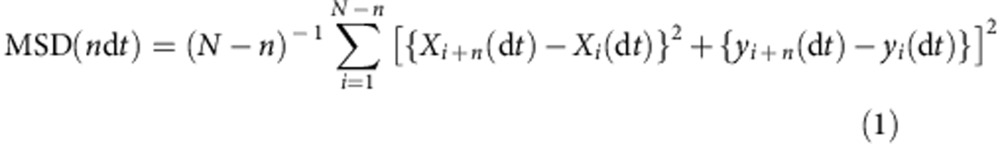


*x*_*i*_ and *y*_*i*_ are the coordinates of an object on frame *i*; *N* is the total number of steps in the trajectory; d*t* is the time interval between two successive frames; and *n*d*t* is the time interval over which displacement is averaged. The diffusion coefficient *D* was calculated by fitting the first two to five points of the MSD plot versus time with equation (2).


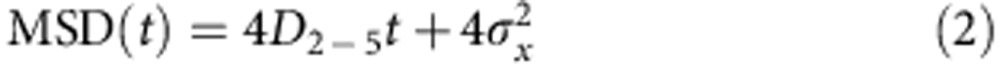


*σ*_*x*_ is the spot localization accuracy in one direction^9^. Given the resolution, trajectories with *D*<10^4^ μm^2^ s^−1^ for QDs were classified as immobile. The size of the average confinement area was calculated fitting the average MSD plot with the equation proposed in ref. 49. Dwell time was calculated as described^49^.

### Electrophysiology in cultured hippocampal neurons

Voltage-clamp experiments were performed on hippocampal neurons (DIV 10 to 12) in the whole-cell configuration of the patch-clamp technique, 24–48 h after transfection of pEGFP (control), RdxT564A–GFP (RdxT564A) or RdxT564D–GFP (RdxT564D). Membrane currents were recorded with an EPC-9 patch-clamp amplifier (HEKA, Lambrecht/Pfalz, Germany). Data were low-pass filtered at 2.9 kHz. Experiments were done at room temperature (21–23 °C). Pipettes were made from borosilicate glass capillaries and had a resistance of 3.0–4.0 MΩ, when filled with intracellular solution. Holding potential was −70 mV. Transient current events were detected by Mini Analysis (version 6.0.1, Synaptosoft, Fort Lee, NJ). For further analysis Igor (Wavemetrics, Portland, OR) and Excel (Microsoft, Redmond, WA) were used. Events with 10–90% rise time larger than 50% decay time were excluded. Tonic currents were defined as bicuculline-sensitive tonic currents.

The extracellular solution contained: 143 mM NaCl, 5 mM KCl, 0.8 mMgCl_2_, 1 mM CaCl_2_, 10 mM HEPES, 5 mM glucose and 0.5 μM TTX, 10 μM CNQX and 50 μM APV, pH adjusted to 7.3 with NaOH. The pipette solution contained: 140 mM CsCl, 1 mM CaCl_2_, 1 mM MgCl_2_, 11 mM EGTA, 5 mM HEPES; pH adjusted to 7.2 with CsOH. Except where indicated all chemicals were purchased from Sigma.

### Electrophysiology in acute hippocampal slices

Thin coronal slices (250–300 μm) were cut from the cortex of postnatal day 21–40 WT (+/+) or *Rdx*-KO (−/−) mice using a Leica T1200s vibroslicer. Slices were cut in an artificial cerebrospinal fluid (CSF) solution containing: 130 mM NaCl, 15 mM KCl, 0.05 mM EGTA, 20 mM HEPES, 4 mM Na-pyruvate and 25 mM glucose, bubbled with 95% O_2_ and 5% CO_2_ at 4 °C. Slices were incubated at 35 °C for 45 min before being cooled to room temperature before use. Over this time, the solution was slowly changed to standard artificial CSF and slices were perfused in the recording bath containing: 125 mM NaCl, 2.5 mM KCl, 1.25 mM NaH_2_PO_4_, 26 mM NaHCO_3_, 2 mM CaCl_2_, 1 mM MgCl_2_ and 25 mM glucose, bubbled with 95% O_2_ and 5% CO_2_. Cells were continuously superfused with 10 μM CNQX and 20 μM AP-5 to block glutamate-mediated fast synaptic transmission. Whole-cell patch-clamp recordings were made from single cells (*V*_H_=−70 mV) using an Axopatch 200B amplifier in the voltage-clamp configuration (Molecular Devices, Sunnyvale, CA). Patch pipettes (3.5–5 MΩ) were filled with solutions containing the following: 120 mM CsCl, 1 mM CaCl_2_, 11 mM EGTA, 10 mM HEPES, 1 mM Mg_2_ATP, 33 mM TEA-OH and 2 mM ATP, pH 7.3. Currents were filtered at 5 kHz (eight-pole Bessel filter) and analysed using Clampex 10.2 (Molecular Devices). Single exponential decay times for mIPSCs were fit using Mini Analysis.

Negative binomial regression analysis was performed to investigate the effects of control versus RdxT564A expression and WT (+/+) versus *Rdx*-KO (−/−) for frequency of occurrence of decay times, 10–90% rise times, amplitudes or IEI and their interaction on the number of events. Levels of significance were set to *P*<0.05, two sided. No adjustment for multiplicity was applied. Calculations were performed using the software Stata (Version 11.2, StataCorp. 2009. Stata Statistical Software: Release 11. StataCorp LP, College Station, TX). Average IEI, decay time, 10–90% rise time and amplitude are arithmetic means of geometric means of each experiment. Student́s *t*-test (two sided) was performed between control versus RdxT564A and WT (+/+) versus *Rdx*-KO (−/−) experiments.

### Production and titration of rAAV vectors serotype 5

Production and titration of rAAV particles was performed by the Hamburg Center for Experimental Therapy Research (HEXT) Vector Core Unit, University Medical Center Hamburg-Eppendorf, Hamburg, Germany.

AAV5 pseudotyped viruses were generated by co-transfection of HEK293-AAV cells (Biocat, Heidelberg, Germany) with the pAAV-Rdx-WT–GFP or pAAV-RdxT564A–GFP pdsAAV-TNNT2 transfer plasmid and the AAV packaging plasmid pDP5rs, which provides the rep2 and cap5 genes and adenoviral helper functions^50^. Generation of recombinant AAV5 particles was carried out as described^51^ with some modifications. HEK293-AAV cells were cultivated in Dulbecco's modified Eagle's medium (high glucose) supplemented with 10% (vol/vol) heat-inactivated foetal calf serum, 0.1 mmol l^−1^ MEM non-essential amino acids, 2 mmol l^−1^ l-glutamine, 100 U per ml penicillin and 100 μg per ml streptomycin. Tissue culture reagents were obtained from Life technologies. Briefly, 6 × 10^6^ HEK293-AAV cells were seeded on 15-cm plates and transfected with polyethylenimine. After 72 h, cells were harvested, washed with PBS and resuspended in AAV-Lysis buffer (100 mM Tris pH 7.6, 166 mM NaCl, 100 mM MgCl_2_). After three freeze–thaw cycles, benzonase (Merck; final concentration 250 U ml^−1^) was added and the lysates were incubated for 1 h at 37 °C. Cell debris was pelleted and vector-containing lysates were purified using iodixanol step gradients. Subsequently, the virus containing solution was re-buffered in D-PBS (#14190169, Thermo Fisher Scientific, Carlsbad, CA), concentrated using centrifugal filter units (Amicon Ultra-4, 40 mwco, Millipore, Darmstadt, Germany) and stored in aliquots at −80 °C until it was used.

The genomic titres of DNase-resistant recombinant AAV particles were determined by quantitative PCR using the SYBR Green qPCR Master MIX 2 (Fermentas, Darmstadt, Germany) and an ABI PRISM 7900HT cycler (Applied Biosystems, Foster City, CA). Vectors were quantified using primers specific for the *TNNT2* promoter sequence. Real-time PCR was performed in a total volume of 10 μl with 0.3 μmol l^−1^ for each primer. pdsAAV–GFP plasmid was used as a copy number standard. A standard curve for quantification was generated by serial dilutions of the respective plasmid DNA. Calculations were done using the SDS 2.4 software (Applied Biosystems).

Physical titres (vector genomes per millilitre (vg per ml)) were estimated at 1.50 × 10^12^ vg per ml for the rAAV5 vector preparations used in the present study.

### Stereotaxic intra-hippocampal injections of rAAV vectors

General anaesthesia induction was achieved with isoflurane (4.0% (v/v)) and O_2_ (0.5 l min^−1^) followed by isoflurane (1.5% (v/v)) and O_2_ (0.5 l min^−1^) during the course of the procedure. The mice also received s.c. application of buprenorphine (0.05 mg per kg in 0.9% saline) to minimize pain and inflammation risk.

The animal's head was positioned and fixed in a stereotaxic frame (digital lab standard frame, 51950, Stoelting instruments, Wood Dale, IL). The head was adjusted to ensure that the Bragma and Lambda positions were in the same horizontal plane prior to infusions of the rAAV vectors through a 2-μl syringe (Neuros Model 7002, Hamilton, Reno, NV). One microinjection of 2 μl was made per hemisphere, targeting the dorsal hippocampus. The coordinates were based on the mouse brain stereotaxic atlas^52^. The coordinates (anterior–posterior (AP) from Bregma, medio-lateral (ML) measured from midline of central sinus, and dorso-ventral (DV) from the dura surface) were as follows: −2.0 mm AP, ±1.8 mm ML, and −2.0 mm DV. A small hole was drilled through the skull (0.45 mm Micro-Drill; Stoelting Instruments) before the cannula was inserted into the area-of-interest and left for 5 min before vector delivery. Stepwise delivery (1 μl) of the viral vector with an interval time of 5 min was performed by retracting the cannula to −1.8 mm (DV) to achieve optimal vector spread in the hippocampus. The vector was delivered at 0.2 μl min^−1^. At the end of infusion, the cannula was left in place for 5 min, retracted to −1.6 mm (DV) and left in place for an additional 2 min before being slowly withdrawn from the brain. Prior to injection into the other hemisphere, the animal received an s.c. injection of Carprofen (5 mg kg^−1^) for post-operation analgesia. After surgery, the cut in the scalp was closed using VetBond (3 M Animal Care Products, St Paul, MN), the animal was taken off of isoflurane and removed from the stereotaxic frame. For immediate recovery, the animal was placed in a clean cage for 1 h placed on a heating mat.

### Behavioural experiments

Subjects: naïve age-matched, 14-week-old male and female, *Rdx*-KO (−/−) and WT (+/+) mice, were bred in a specific-pathogen-free breeding facility in the Institute of Molecular Neurogenetics (Center for Molecular Neurobiology (ZMNH), University Medical Center Hamburg-Eppendorf, University of Hamburg). 129S4/SvJae mice were backcrossed with C57BL/6J over seven generations to reveal a defined genetic background (for details of genetic construction, breeding and subsequent genotyping, see ref. 28). Mice were ∼14 weeks old at the beginning of the behavioural analysis. Mice were weaned at postnatal day P21 and housed in groups of littermates separated by sex (2–4 individuals per cage). They were maintained in an acclimatized animal vivarium (21±1 °C, relative humidity at 55±5%) under a reversed light/dark cycle. Rooms were illuminated between 1900 and 0700 hours. Mice had *ad libitum* access to food and water and were tested during the dark cycle.

The rotarod task, the inverted grid task and the Y-maze spatial recognition task (I) were performed at the laboratory of Behavioural Neurobiology at the Swiss Federal Institute of Technology Zurich. Here one cohort of subjects was used. The testing sequence was: (1) rotarod task (WT (+/+)=8, *Rdx* (−/−)=8); (2) inverted grid task (WT (+/+)=8, *Rdx* (−/−)=8); (3) Y-maze spatial recognition task 3 min ITI (WT (+/+)=8, *Rdx* (−/−)=8). The experiments were approved by the Swiss Cantonal Veterinary Office in accordance to the ethical standards required by the Swiss Act and Ordinance on Animal Protection, the European Council Directive 86/609/EEC and the National Institutes of Health Guide for Care and Use of Laboratory Animals (National Research Council, 1996).

### Accelerating rotarod test

The Ugo-Basile accelerating rotarod for mice (model: 7650; Comerio, VA, Italy) was used. Two subjects were tested concurrently and placed on the rotating drum at a baseline speed of 4 r.p.m. Speed was increased linearly to 40 r.p.m. over the 5-min testing period. Latencies to fall off were recorded, with a maximum of 300 s.

### Inverted grid test

A standard wire cage lid was used, with the perimeter walled off by masking tape to prevent the mice from walking off the edge. To begin the test, mice were placed in the centre of the wired area. The cage lid was then gradually turned 180° until the animal was hanging upside down at 40 cm above a soft landing surface. The time until the mice fell off was recorded (maximum: 300 s).

### Novelty preference test in the Y-maze (I)

Eight WT (+/+) and eight *Rdx*-KO (−/−) mice including both sexes were tested in the Y-maze as described below. Apparatus: novelty preference tests were assessed using a grey, wooden Y-maze (80 cm above the ground) located in the middle of a room containing a variety of extra maze cues. All arms were identical (length 50 cm; width 10.5 cm; spaced at 120°). The entire Y-maze was enclosed by a 10-cm transparent wall (thickness 1 cm). The floor of the maze was covered with sawdust bedding. Images were captured with a digital camera at 5 Hz and analysed with the Ethovision tracking system (Version XT 8.5, Noldus Technology, Wageningen, Netherlands) for automated track path analysis. Procedure: the subjects were exposed to two arms (start and sample arm) during the first phase of the test (sample phase). The remaining third arm constituted the novel arm during the subsequent test phase (novelty preference test; see Fig. 7e). Allocation of sample and novel arms were counterbalanced within each experimental group. During the training phase the entrance to the novel arm was blocked by a grey wooden door (high 12 cm, wide 7.5 cm, thickness 0.5 cm). Mice were placed at the end of the start arm and allowed to freely explore both the start and the sample arm. The sample phase lasted 5 min starting from the time when the animal left the start arm and entered the sample arm for the first time. The test phase was conducted after a rest period of 3 min spent in the waiting cage. It began with the mouse being placed at the end of the start arm with free access to all three arms of the Y-maze. It lasted for 3 min starting from the time when the animal left the start arm. In the course of the novelty preference test (test phase), the former sample arm is termed the familiar arm (see Fig. 7e). During sample and test phase intervals, the sawdust from each arm was replaced to avoid any olfactory cues. Preference for the novel arm was calculated as (time spent in the novel arm per time spent in all arms) × 100%.

### Behavioural tasks including α5IA or rAAV

All other behavioural tasks, including the response to systematic GABA_A_R-α5 inverse agonist (α5IA) and the rAAV-mediated hippocampal expression of different Rdx-mutant proteins, were performed at the University Medical Center Hamburg-Eppendorf, Center for Molecular Neurobiology, ZMNH. For experiments including α5IA, three cohorts of animals were used. The testing sequence was: cohort A: (1) elevated plus maze (WT (+/+)=29, *Rdx* (−/−)=28); (2) open field (WT (+/+)=29, *Rdx* (−/−)=28); (3) Morris water maze with systematic α5IA (WT (+/+) veh=15; WT (+/+) α5IA=14; *Rdx* (−/−) veh=13; *Rdx* (−/−) α5IA=15). Cohort B: Y-maze spontaneous alternation (WT (+/+)=14, *Rdx* (−/−)=11). Cohort C: Y-maze spatial recognition task 1 h ITI with systematic α5IA (WT (+/+) veh=11), *Rdx* (−/−) veh=11), WT (+/+) α5IA=12) and *Rdx* (−/−) α5IA=12)). For the Morris Water Maze experiment including rAAV-mediated gene expression, a cohort comprising 21 male mice received bilateral hippocampal microinjections of either the control (rAAV–eGFP) viral vectors [7 Rdx-WT (+/+) mice], Rdx-WT expressing (rAAV-Rdx-WT–eGFP) vectors [7 Rx-KO (−/−) mice] or RdxT564A expressing (rAAV-Rdx-T564A–eGFP) vectors [7 Rdx-WT (+/+) mice]. After surgery, mice were housed in single cages. We had previously established in a pilot study that stable transgene expression, restricted to the hippocampus formation, peaked at 5 weeks post microinjection. Hence, behavioural experiments commenced 6 weeks post surgery at 14–16 weeks of age. All experiments were conducted in accordance with the German and European Community laws on protection of experimental animals and were approved by the local authorities of the City of Hamburg (Behörde für Soziales, Familie, Gesundheit und Verbraucherschutz, Lebensmittelsicherheit und Veterinärwesen, Billstraße 80, 20539 Hamburg, Germany, reference 106/10).

### Elevated plus maze

The elevated plus maze was made of water proof polyvinyl foam material and contained a removable white plastic floor. It was elevated 70 cm above the floor and positioned in one corner of the testing room with diffuse dim lighting (25 lux) in the open arms. The maze consisted of four equally spaced arms (length × width: 30 × 5 cm) radiating from a central square 5 × 5 cm. One pair of opposing arms was enclosed with opaque walls, whereas the remaining two arms were exposed with a 2 mm high perimeter border along the outer edges. A digital camera located above the maze captured images, which were transmitted to a PC running the Ethovision XT8.5 tracking system. For each trial, the mouse was gently placed in the central square facing one of the open arms. It was allowed to explore freely for 5 min before being gently removed and returned to the home cage. Two measures were scored as follows: (i) Percentage time spent in the open arms (open per (open+closed) × 100%). (ii) Total distance travelled in the 5-min period.

### Y-maze continuous spontaneous alternation

Spontaneous alternation behaviour was evaluated in a symmetrical Y-maze consisting of beige plastic opaque walls (9(W) × 39(D) × 16(H) cm, spaced at 120°). The experiment was performed in a room with dim lighting (10 lux). Subjects were gently placed in the distal part of one arm and were allowed free access to the three arms for 5 min. Arm entry was recorded once the mouse moved beyond the central triangle and entered one of the arms. The sequence of arm visits was recorded manually by the observer. An alternation was scored when the mouse made three successive arm entries into each of the three arms. Non-overlapping three entries were counted. Percentage alternations were calculated as follows: (actual alternations per maximum possible alternations) × 100%. Subsequently, the mice were gently removed from the maze and returned to their home cage. The maze was cleansed with 30% ethanol, water and dried thoroughly between trials.

### Spontaneous open field activity

Mice were tested in a box consisting of four identical arenas (length × width × height: 50 × 50 × 50 cm) made of water proof closed polyvinyl foam material. Two arenas were designated for testing female mice, and the other two for testing male mice. The arenas were positioned below a metallic stand consisting of four equally distributed lamps that provided diffuse (50 lux) lighting in each arena. Exploratory behaviour was recorded by a digital camera mounted in the middle of the metallic stand and directly above the four arenas. Images were captured and transmitted to a PC running the Ethovision tracking system.

Mice were introduced to the centre of the Arena and allowed to explore undisturbed for 60 min, and the test was repeated over 2 consecutive days. Between trials, the arenas were cleansed with soapy water and 30% EtOH. Horizontal locomotor activity was indexed as distance traveled (path length) in 10-min consecutive bins.

### Analysis of PERM levels after open field exposure in mice

Hippocampal PERM protein levels were analysed in a cohort of 12 age-matched 16-weeks-old male C57Bl6 mice. Three groups of mice were killed either before, following and 24 h after testing in the open field. To quantify PERM protein levels, subjects were killed by cervical dislocation and the brain was removed. The hippocampus was dissected in ice cold PBS containing 50 mM NaF (Sigma) and was subsequently transferred into lysis buffer containing: 1% (v/v) Triton X-100, 2 mM EDTA, 1 mM PMSF (Applichem), PhosSTOP phosphatases inhibitors and Complete proteases inhibitors (Roche) in PBS. The tissue was homogenized using a 26-gauge needle. After incubation for 1 h at 4 °C, lysates were centrifuged at 1,000*g* for 10 min. The resulting supernatant was boiled in SDS sample buffer after adjustment of protein concentrations using a BCA assay. Samples were analysed by WB.

### Novelty preference test in the Y-maze (II)

Here, four treatment groups were tested in a clear symmetrical Y-maze as described below. Apparatus: the apparatus consisted of a symmetrical Y-maze (80 cm above the ground) located in the middle of a room containing a variety of extra maze cues. All arms were identical (length 50 cm; width 10.5 cm; spaced at 120°). The entire Y-maze was enclosed by a 10 cm transparent wall (thickness 1 cm) and contained a white rough floor insert. Images were captured with a digital camera at 5 Hz and analysed with the Ethovision tracking system. Procedure: subjects were injected (i.p.) with either 5 mg per kg α5IA (for drug administration, see below) or vehicle solution (WT (+/+)-veh (*n*=11), *Rdx* (−/−)-veh (*n*=11), WT (+/+)-α5IA (*n*=12) and *Rdx* (−/−)-α5IA (*n*=12)). After drug administration, the mice were individually kept in standard Makrolon cages (26 × 21 × 14 cm) and left undisturbed for 30 min prior to testing.

The testing procedure is as described above (see novelty preference test in the Y-maze I). Here the delay interval (ITI) between training phase and the novelty preference test (test phase) was extended to 60 min. Mice were individually kept in Makrolon cages and left undisturbed in an adjacent room during the delay interval. The maze was cleansed with 30% ethanol, water and dried thoroughly between test phase intervals and trials. Preference for the novel arm was calculated as (time spent in the novel arm per time spent in all arms) × 100%.

### Reversal learning in the Morris water maze

About 57 mice (29 WT (+/+) and 28 *Rdx*-KO (−/−)) including both sexes and 21 male mice (7 *Rdx* (+/+) rAAV-Rdx-T564A; 7 *Rdx* (−/−) rAAV-Rdx-WT; 7 *Rdx* (+/+) rAAV-control), respectively, were trained in the Morris water maze experiment using a circular water tank (diameter 1.5 m, height 75 cm; water depth 35 cm, 22±1 °C). The water was made opaque by the addition of white non-toxic tempera paint to hide the escape platform. The tank was located in the middle of a test room enriched with distal visual cues. Water was replaced every other day. Four equally spaced cardinal points (N, S, E and W) were designated around the pool to serve as starting points where the animals were placed into the water. The pool area was divided into four virtual quadrants (NE, SE, SW and NW) of equal size. The escape platform was made of transparent Plexiglas disc (diameter 14 cm) and mounted on top of a solid Plexiglas tube, which could be fixed to the bottom of the pool by a solid metallic block. Images were captured with an analogue camera (SSC-DC58AP, Sony) and analysed with the Ethovision tracking system for automated swim path monitoring. Visible platform training: Prior to hidden platform training, the mice were tested on the cued version of the task whereby the location of the submerged platform was indicated by a salient local cue visible above the water level. Subjects received eight cued trials in total, conducted across 2 consecutive days. On each day, the mice received four consecutive trials (ITI=8 min) to navigate to the visible platform. The release point (S and N) for each day was constant across trials, but the location of the visible platform was varied in a pseudo-random manner across trials. It was positioned in the middle of the three quadrants and the centre of the pool. The quadrant designated for hidden platform training was not used. A trial ended when the mouse escaped onto the platform or after 60 s had elapsed, at which point it was guided to the platform by the experimenter. The mouse was allowed to remain on the platform for 15 s before being retrieved and briefly dried with a towel. It was returned to a waiting cage and kept warm by a heating pad until the next trial commenced. Drug administration: α5IA (ORGA-Link, Magny-Les-Hameaux, France) was dissolved in DMSO, Cremophor EL (Sigma) and water (10:15:75). Henceforth and on each day, the mice were injected (i.p.) with either 5 mg kg^−1^ α5IA or vehicle solution ((+/+)veh=15; (+/+)α5IA=14; (−/−)veh=13; (−/−) α5IA=15). After drug administration, the mice were individually kept in standard Makrolon cages and left undisturbed for 30 min prior to testing in the water maze. Reference memory acquisition: on days 3–8, the animals were trained to locate an escape platform located 0.5 cm below the water surface. It was constantly located in the middle of the target quadrant, which was counterbalanced across subjects. The α5IA-injected mice received four trials per day and the mice with bilateral hippocampal rAAV-Rdx expression two trials per day (ITI=5 min) with the starting positions (N, E, S and W) varying in a pseudo-random sequence. A trial ended when the mouse successfully climbed onto the platform or after 60 s had elapsed, during which it was gently guided to the platform by the experimenter. Retention probe test: on day 9, a 60-s probe trial was conducted. The platform was removed from the pool, and the mice gently released from the quadrant located directly opposite the target quadrant. They were allowed to swim for 60 s, then gently removed from the pool and dried down with a towel. They were left in heated Makrolon cages until completely dry before being returned to their home cages. Performance was assessed by percentage path length (distance moved) in the target quadrant. Reversal learning test: from day 10 onwards, the hidden platform was switched to the opposite quadrant, and the mice received additional training as described in the RM acquisition.

### Statistical analysis

The sample size was adjusted according to results of prior pilot data sets or studies that used similar methods or paradigms. Power estimation was performed using G*Power 3 (Version 3.1.3, Düsseldorf, Germany). Data were analysed using SPSS (PASW) Statistics 19.0 (Version 19). Prior to analysis, SPSS was used to explore the data, estimate and compare the variance within each group. Appropriate statistical tests were then chosen. The statistical tests used are outlined in the corresponding figure legends for each experiment. A two-tailed type I error rate of *P*<0.05 was adopted as a yardstick for statistical significance. The analyses included the between-subjects factors: genotype (*Rdx*-KO (−/−) versus WT (+/+)), sex (female versus male), treatment (vehicle versus α5IA) and repeated measures (within subjects) factors as appropriated by the design of the experiment. These included factors such as arms, bins and days. The initial analyses provided no evidence for main effects or interactions involving sex. Hence, the factor of sex was dropped from the analyses to increase statistical power. Supplementary restricted analyses were also performed to assist data interpretation whenever appropriate. *Post-hoc* comparisons were performed with Bonferroni correction based on the overall error variance associated with the relevant factor. During the experiment, the investigator was blinded to the genotype or the treatment identity of the subjects. All biological experiments were repeated at least three times using independent cell cultures or individual subjects (biological replications).

## Author contributions

T.J.H. performed and analysed the biochemical and immunochemical experiments and performed the behavioural experiments with the help of P.B., S.D., B.K.Y., J.F. and S.T. M.M. performed and analysed the behavioural experiments using α5IA. K.G. and A.T. performed and analysed the single particle tracking experiments. W.H., P.T., T.G.S. and J.R.S performed and analysed the electrophysiological experiments; L.H. performed the additional statistical analysis. T.J.H. and M.K designed the study, analysed and interpreted the data and wrote the manuscript with the help of M.M., F.F.H. and J.R.S.

## Additional information

**How to cite this article:** Hausrat, T. J. *et al*. Radixin regulates synaptic GABA_A_ receptor density and is essential for reversal learning and short-term memory. *Nat. Commun.* 6:6872 doi: 10.1038/ncomms7872 (2015).

## Supplementary Material

Supplementary Information Supplementary Figures 1-8 and Supplementary References
