# Supplementary material for: Radixin regulates synaptic GABAA receptor density and is essential for reversal learning and short-term memory
Source: Nat Commun. 2015 Apr 20;6:6872. doi: 10.1038/ncomms7872 (PMC4411296; doi:10.1038/ncomms7872)
Supplement: Supplementary Information — Supplementary Figures 1-8 and Supplementary References [file ncomms7872-s1.pdf]

Supplementary Information

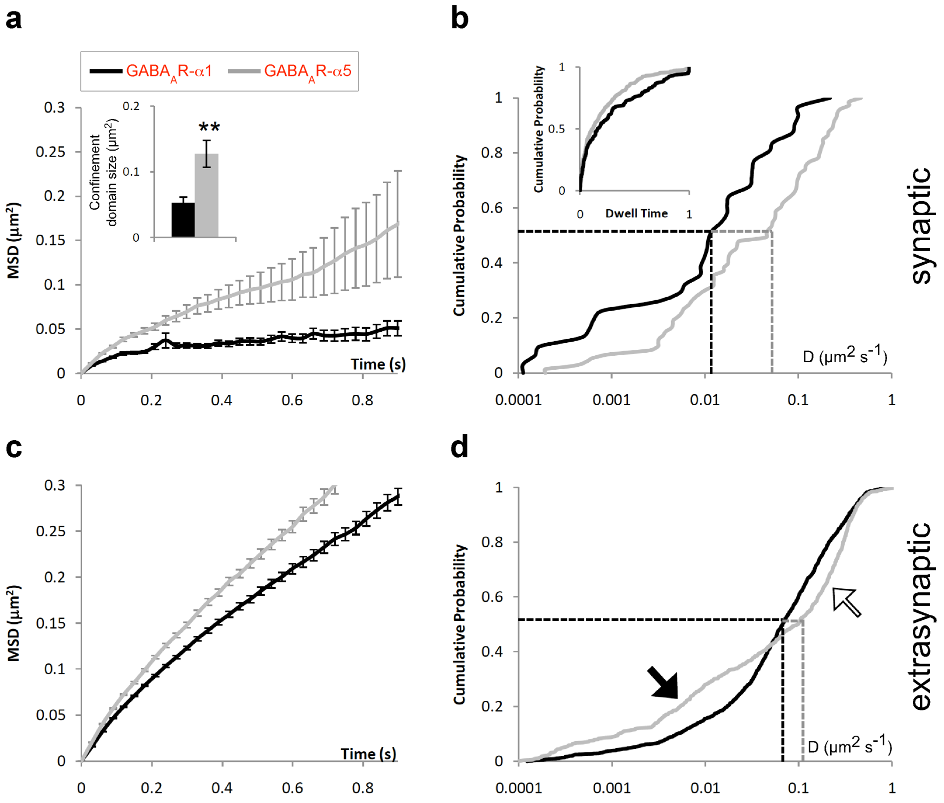

Supplementary Figure 1. Diffusion properties of GABA$_A$R-α1 and GABA$_A$R-α5. Supplementary data related to Figure 1. (**a-b**) Characterization of diffusion at inhibitory synapses. (**a**) Mean square displacement (MSD) versus time plot for GABA$_A$R−α1 (black) and GABA$_A$R−α5 (grey) quantum dot (QD) trajectories at gephyrin-positive clusters. Note the negatively bent curves indicating a confined diffusion. Inset: The average size of the confinement domain for GABA$_A$R−α1 (black, 0.053±0.008 µm^2) versus GABA$_A$R−α5 (grey, 0.127±0.020 µm^2, p<0.01, t-test) at synapses. (**b**) Cumulative probability plot of GABA$_A$R−α1 and GABA$_A$R−α5 QD diffusion coefficients at gephyrin-positive synapses (p<0.05, KS test). Median diffusion coefficients for GABA$_A$R−α1 (0.012 µm^2 s^{-1}) and GABA$_A$R−α5 (0.046 µm^2 s^{-1}) are indicated by dashed lines. Inset: cumulative probability of dwell time (time at synapses per total time). (**c-d**) Characterization of diffusion outside of inhibitory synapses. (**c**) MSD versus time plot for GABA$_A$R−α1 (black) and GABA$_A$R−α5 (grey) QD trajectories. Note the linear curves characteristic of Brownian movements. (**d**) Cumulative probability plot of GABA$_A$R−α1 and GABA$_A$R−α5 QD diffusion coefficients (p<0.001, KS test). Median diffusion coefficients for

$GABA_AR{-}\alpha1$ (0.067 $\mu m^2 s^{-1}$) and $GABA_AR{-}\alpha5$ (0.097 $\mu m^2 s^{-1}$) are indicated by dashed lines. $GABA_AR{-}\alpha5$ diffusion coefficient display a bi-modal distribution with a population with lower (black arrow) and another one with higher (open arrow) diffusion coefficients than $GABA_AR{-}\alpha1$. Note that the distribution of the latter is unimodal. Error bars: mean±s.e.m.

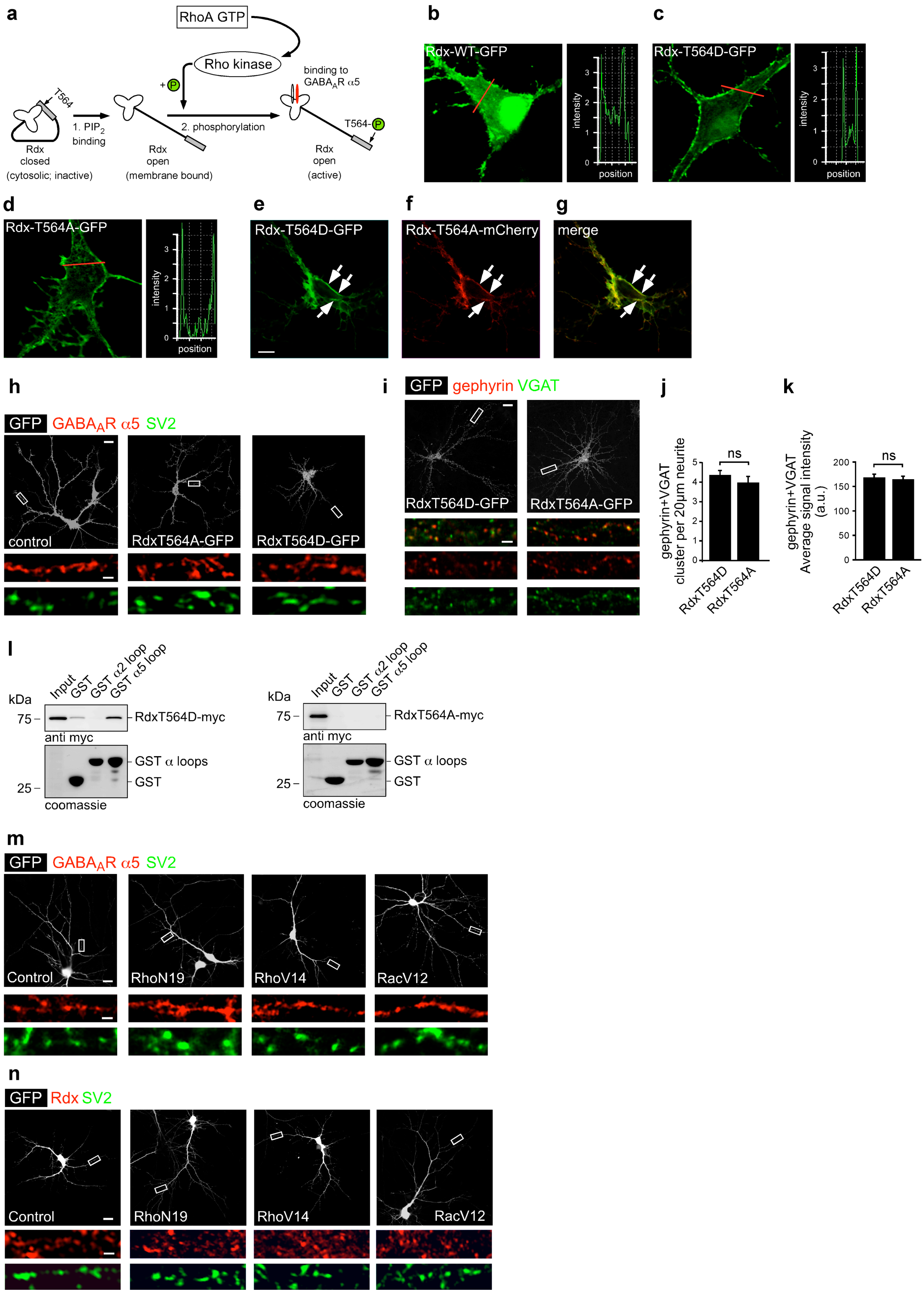

Supplementary Figure 2. Supplementary data related to Figure 2. (**a**) Radixin (Rdx) exists in two structural conformations: a closed inactive (cytosolic localization), and an open active form (plasma membrane bound)[1]. Activation of Rho-dependent kinase through RhoA GTPases leads to the phosphorylation of Rdx at a conserved threonine residue at position 564 (T564) (Fig. 2f-g and 3a-c, main text). T564 phosphorylation induces a conformational change that facilitates the binding of Rdx

to $GABA_AR$-α5 (Fig. 2e and Supplementary Fig. 2l), leading to altered synaptic or extrasynaptic localization of $GABA_AR$-α5, respectively (Fig. 2a-b, Fig. 2h-i and Fig. 9, main text). (**b-d**) Analysis of single amino acid substitutions within Rdx: RdxT564 to aspartic acid (RdxT564D-GFP; phospho-mimicking mutant) or alanine (RdxT564A-GFP; phospho-minus mutant) compared to GFP-fusion proteins of wild type Rdx (RdxWT-GFP) according to their distribution within the cell. Line scan analyses were used to measure GFP signal intensities after expression in hippocampal neurons. The red line indicates fluorescent intensities over the crossed sections plotted in the corresponding graphs. Intensity peaks are reduced in the cytosol for RdxT564D, as well as for RdxT564A compared to wildtype controls. Both Rdx mutants show increased localization at the plasma membrane compared to wildtype control. (**e-g**) Subcellular distribution and colocalization analysis of RdxT564D-GFP and RdxT564A-mCherry fusion proteins after overexpression in hippocampal neurons. Arrows depict colocalization of RdxT564D-GFP and RdxT564A-mCherry at the plasma membrane. This indicates enriched localization of both RdxT564D and RdxT564A fusion proteins at the plasma membrane. Therefore, upon overexpression both RdxT564D or Rdx564A compete with endogenous Rdx at the plasma membrane. Scale bar: 20 µm. (**h**) Immunostainings of endogenous $GABA_AR$-α5 (red) and the pre-synaptic marker protein SV2 (green) upon expression of GFP (grey, control), RdxT564D-GFP (grey) (RdxTD) or RdxT564A-GFP (grey) (RdxTA) in primary hippocampal neurons. The boxed dendritic regions are shown at higher magnification. Merged images are shown in Figure 2a (main text) to indicate synaptic localization of $GABA_AR$-α5 upon colocalization with SV2. (**i-k**) Analysis of the density of inhibitory synapses after expression of RdxT564A-GFP or RdxT564D-GFP in primary hippocampal neurons. (i) Immunostainings of endogenous gephyrin (red) and the inhibitory pre-synaptic marker protein VGAT (green) after expression of RdxT654D-GFP or RdxT564A-GFP, as indicated (grey). The boxed dendritic regions are shown at higher magnification for single channels and merged images to depict co-localization of endogenous gephyrin and VGAT. The quantification of (j) cluster density per 20 µm dendrite (RdxT564D: 4.30±0.23, RdxT564A: 3.93±0.27) and (k) average signal intensities (a.u.) (RdxT564D: 166.51±5.10, RdxT564A: 162.86±4.86) of co-localized gephyrin and VGAT cluster revealed no differences upon RdxT564A and RdxT564D overexpression (ANOVA; n=3 with 16-24 cells per experiment, error bars: mean±s.e.m.). This indicates no change in the density of inhibitory synapses after expression of RdxT564A-GFP. Error bars: mean±s.e.m. (**l**) GST pull-down assays detect binding of RdxT564D, but no binding of RdxT564A to the $GABA_AR$-α5

cytosolic loop. The $GABA_AR$-$\alpha2$ cytosolic loop displayed no binding to RdxT564D or to RdxT564A, respectively (*n*=3). (**m**) Immunostainings of endogenous $GABA_AR$-$\alpha5$ (red) and the pre-synaptic marker protein SV2 (green) after expression of the RhoA GTPases as indicated (grey). The boxed dendritic regions are shown at higher magnification. Merged images are shown in Figure 2h (main text) to indicate synaptic co-localization of $GABA_AR$-$\alpha5$ and SV2. (**n**) Immunostainings of endogenous Rdx (red) and the pre-synaptic marker protein SV2 (green) upon expression of the RhoA GTPases, as indicated (grey). The boxed dendritic regions are shown at higher magnification. Merged images are shown in Figure 2j (main text) to indicate synaptic co-localization of Rdx and SV2. Scale bars: 30 µm and 3 µm (magnifications), respectively.

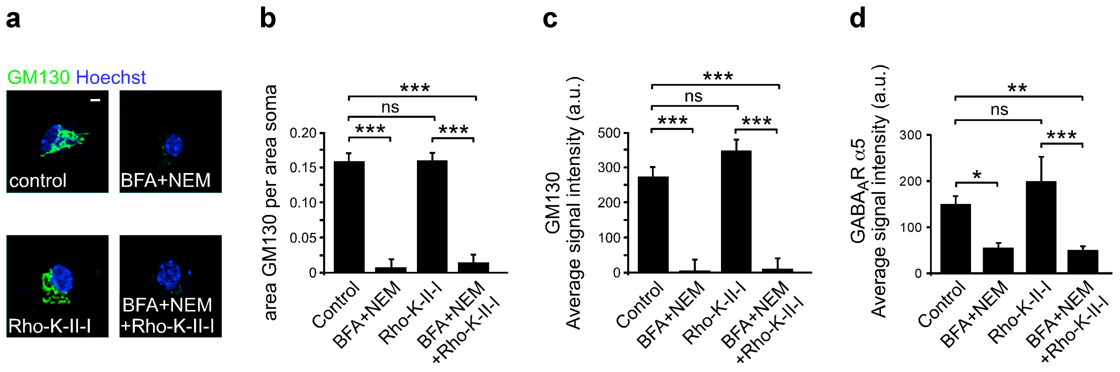

Supplementary Figure 3. BFA and NEM decrease GM130 and GABA$_A$R-α5 signal intensities independent of Rho kinase inhibition. Supplemental data related to Fig. 3 d-g analyzing the morphology of the Golgi apparatus and GABA$_A$R-α5 expression levels in hippocampal neurons after application of 0.5 μM brefeldin A (BFA) and 0.5 μM N-ethylmaleimide (NEM) in the presence or absence of Rho-kinase-II inhibitor. BFA is known to disrupt the integrity of the Golgi apparatus, and the export of receptors from the late endoplasmic reticulum[2, 3]. NEM interferes with exocytotic protein delivery[4]. (**a**) Immunostainings of endogenous GM130 (green), a marker protein of the Golgi apparatus, to evaluate the morphology of the Golgi apparatus in somatic regions of hippocampal neurons after treatment with BFA and NEM in the presence or absence of Rho-kinase-II inhibitor. Hoechst staining (blue) was used to detect the nucleus. (**b**) Independent of Rho-kinase-II inhibition, treatment with BFA and NEM significantly reduced the GM130 positive area within the soma (control: 0.16±0.01, BFA+NEM: 0.01±0.01, RhoK-II-I: 0.16±0.01, BFA+NEM+RhoK-II-I: 0.01±0.01). (**c**) This was also reflected by the quantification of GM130 average signal intensities (in a.u., control: 3391.63±339.34, BFA+NEM: 49.71±366.53, RhoK-II-I: 4318.42±366.53, BFA+NEM+RhoK-II-I: 107.92±366.53). Given the high protein turnover of GABA$_A$ receptors[5], blockade of vesicle segregation and exocytosis should reduce the expression levels of the receptors in neuronal dendrites. (**d**) Indeed, GABA$_A$R-α5 average signal intensities were significantly reduced after application of BFA and NEM, but were independent of Rho-kinase-II inhibitor treatment (arbitrary units (a.u.), control: 150.80±16.16, BFA+NEM: 56.92±9.79, RhoK-II-I: 199.81±51.21, BFA+NEM+RhoK-II-I: 51.38±6.87). The previously described synaptic redistribution of GABA$_A$R-α5 upon Rho kinase II inhibitor treatment was still evident, although the overall GABA$_A$R-α5 cluster concentrations were decreased (see Figure 3 f and g). Together this indicates a synaptic redistribution of GABA$_A$R-α5 upon Rho-kinase-II inhibition independent of vesicle segregation and exocytosis. ANOVA and post hoc test were used for

statistical analysis. *n*=3 experiments with 22-26 cells per group. Scale bar: 3 µm. Error bars: mean±s.e.m.

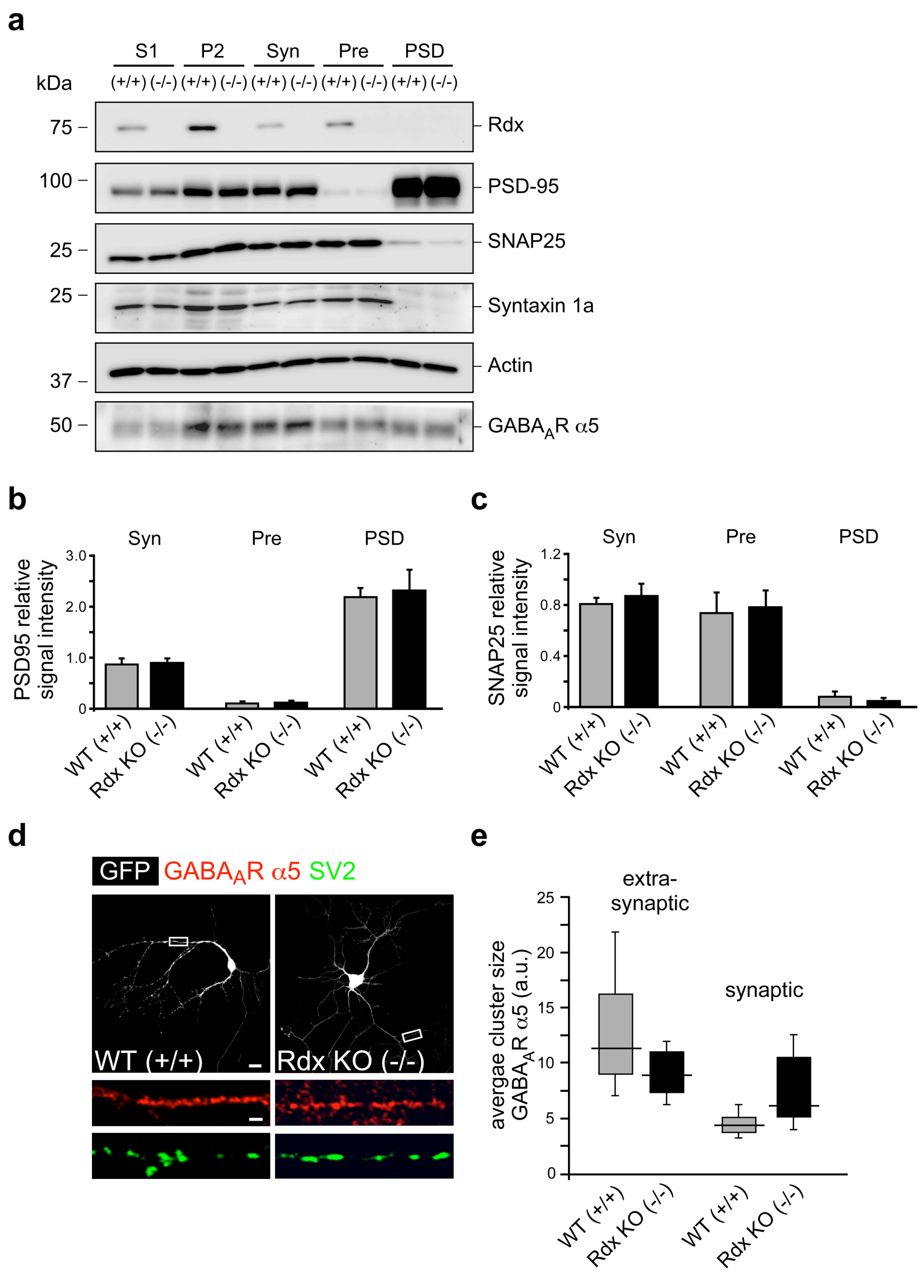

Supplementary Figure 4. Subcellular distribution of GABA$_A$R-α5 in hippocampal neurons of WT (+/+) and *Rdx*-KO (-/-) mice. Supplementary data related to Figure 4. (**a**) Analysis of protein expression levels after differential centrifugation of forebrain lysates derived from WT (+/+) or *Rdx*-KO (-/-) mice. S1: post nuclear supernatant; P2: plasma membrane pellet; Syn: Synaptosoms; Pre: pre-synaptic fraction; PSD: post synaptic density. Representative western blot of three independent experiments using 3 mice per genotype, each. ANOVA was used for statistical analysis. (**b, c**) Quantification of signal intensities of (b) PSD-95, a marker protein of the PSD, and (c) SNAP25, a marker protein of the pre-synaptic active zone[6, 7]. The correct segregation of both marker proteins confirms the purity of the preparation. (**d**) Individual immunofluorescent channels of the images shown in

Figure 4c (main text). Immunostaining of endogenous $GABA_AR$-α5 (red) and the pre-synaptic marker protein SV2 (green) upon expression of GFP (grey) in hippocampal neurons derived from *Rdx* KO (-/-) and WT (+/+) control mice. The boxed dendritic regions are shown at higher magnification. Merged images are shown in Figure 4c (main text) to indicate synaptic colocalization of $GABA_AR$-α5 and SV2. (**e**) Box plots displaying full range variation of the average cluster sizes for $GABA_AR$-α5 at synaptic and extrasynaptic sites in *Rdx*-KO (-/-) mice and WT (+/+) control shown in Figure 4g (main text). Lower und upper borders of each bar indicate the first and third quartile, respectively. Horizontal lines in each bar depict the median. Error bars represent the absolute minimum and maximum, respectively. Analysis of $GABA_AR$-α5 cluster sizes revealed increased clusters at synaptic sites and decreased clusters at extrasynaptic (SV2-negative) sites after radixin depletion in hippocampal neurons (compare with Figures 4f and 4g, main text). Notably, a quantification of pooled synaptic and extrasynaptic cluster sizes revealed no significant differences between both genotypes (see Figure 4h, main text). This suggests a redistribution of $GABA_AR$-α5 within the plasma membrane, rather than a change in receptor expression levels upon radixin depletion. Scale bars: 30 µm and 3 µm (magnifications), respectively.

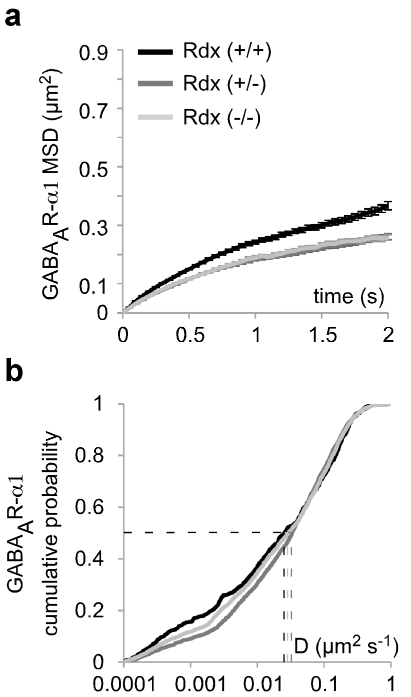

Supplementary Figure 5. Diffusion of $GABA_A$R-α1 in Rdx-depleted hippocampal neurons is unaffected. Supplementary data related to Figure 5. **(a-c)** Diffusion of $GABA_A$R-α1 in hippocampal neurons derived from WT (+/+) (black), *Rdx* (+/-) (dark grey) or *Rdx* (-/-) (light grey) mice. (**a**) Mean square displacement (MSD) versus time plot for $GABA_A$R-α1-quantum dot (QD) trajectories. The average size of $GABA_A$R-α1-QD confinement size revealed no significant differences for *Rdx* (-/-) (0.368±0.010 μm^2) compared to *Rdx* (+/-) (0.377±0.011μm^2) and WT (+/+) (0.355±0.013 μm^2) (p=ns, t-test). (**b**) Cumulative probability plot of $GABA_A$R-α1-QD diffusion coefficients in hippocampal neurons derived from *Rdx* (-/-) (0.029 $\mu m^2 s^{-1}$) compared to *Rdx* (+/-) (0.033 $\mu m^2 s^{-1}$) and WT (+/+) mice (0.026 $\mu m^2 s^{-1}$) (p=ns, KS test). Error bars: mean±s.e.m.

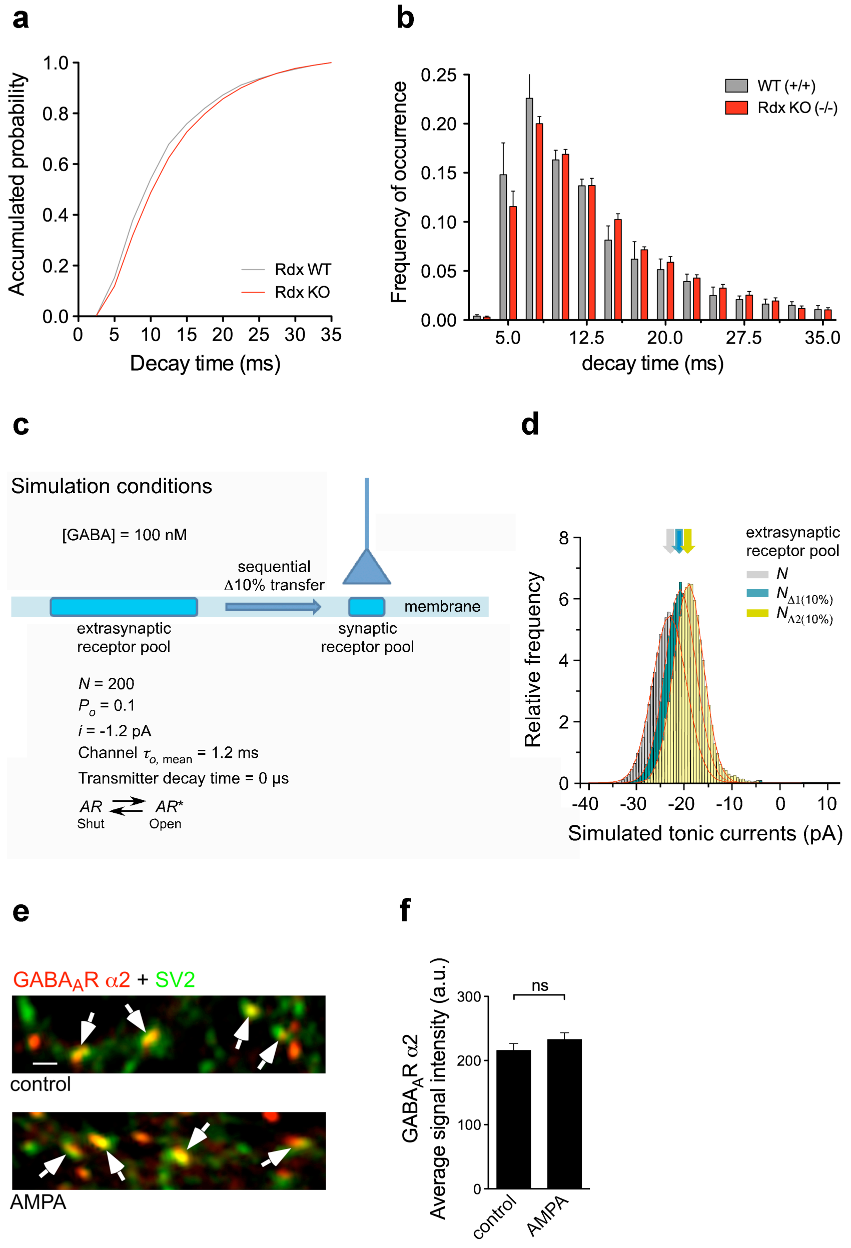

Supplementary Figure 6. Supplementary data related to Figure 6. (**a**) Accumulative probability plot of mIPSC Decay time in hippocampal slices derived from *Rdx* (-/-) and WT (+/+) mice. (**b**) Number of events for indicated decay time intervals (frequency distribution histogram). A negative binominal regression was used for statistical analysis. Average mIPSC for amplitude, 10-90 rise time, 10% decay time and inter event interval (IEI) remain similar across genotypes (Amplitude (pA) WT (+/+): 32.20±1.27; *Rdx* (-/-): 29.07±1.05. Rise time (ms) WT (+/+): 1.17±0.04; *Rdx* (-/-): 1.13±0.03. Decay time (ms) WT (+/+): 9.63±0.78; *Rdx* (-/-): 10.27±0.34. IEI (ms) WT (+/+): 54.63±10.82; *Rdx* (-/-): 53.86±7.37). Data were obtained from *n*=4-8 animals per genotype. (**c-d**) Simulation of GABA tonic currents

during extrasynaptic $GABA_A$ receptor mobility. (c) Schema depicting the assumptions made and the basis of the current simulations where N = number of receptors, P_o is open probability, i = single channel current, $\tau_{o,mean}$ is the mean GABA channel open time, and [GABA] represents GABA concentration. The three simulations are made on the basis of two serial transfers of 10% of extrasynaptic $GABA_A$ receptors to the synaptic compartment. (d) Simulated tonic current amplitude distribution fitted with Gaussian functions. The arrows indicate the mean tonic currents for N = 200 (grey), 180 (cyan) and 162 (yellow) receptors. Our experimental imaging results (see Fig. 2-4) indicated that by preventing the interaction of α5 subunits with radixin, we lost $GABA_AR$-α5 subunits and presumably their associated receptors from the extrasynaptic zone but gained $GABA_AR$-α5 subunit receptors at inhibitory synaptic clusters. In electrophysiological recordings (see Fig. 6 e-k), we observed a modulation of phasic inhibitory currents, but not of tonic inhibitory currents. Therefore we applied a computational simulation reflecting the redistribution of extrasynaptic $GABA_AR$s. In this simulation, several key but plausible assumptions are made, which can also be supported by experimental data[8, 9]. We modelled an extrasynaptic zone containing 200 $GABA_A$ receptors. We further deduced that the GABA concentrations would be low for tonic inhibition (~ 100 nM). The kinetic rate constants for GABA binding and unbinding, and the conformational constants for channel opening and shutting, with GABA channel mean open times ($\tau_{o,\ mean}$) and open probabilities (P_o) are based on our previous single channel work for αβγ subunit-containing $GABA_A$ receptors[8, 9]. The simulations involved creating tonic current fluctuations with initially N = 200 receptors and then repeating when the receptor number was consecutively reduced by 10% twice (thus to N = 180 and 162 receptors, respectively). The GABA current fluctuations over 30 s were collected and analysed as a frequency distribution and then fitted with a Gaussian function. Mean tonic current values are indicated. It is notable that a sequential reduction of 10% in the number of extrasynaptic receptors will give only a shift of < 2 pA in the tonic current, which is likely to be obscured by background noise in real recordings. This concurs with the electrophysiological observations for I_{tonic} (see Fig. 6 i-k). (**e-f**) The synaptic distribution of $GABA_AR$-α2 in cultured hippocampal neurons treated with AMPA is not affected (see Fig. 6t). (e) Immunostainings related to Fig. 6 t: Synaptic co-localization (arrows) of endogenous $GABA_AR$-α2 (red) with the presynaptic vesicle marker SV2 (green) in dendritic regions of neurons treated with 4µM AMPA for 1 h. Average signal intensities for $GABA_AR$-α2 (f) revealed no significant differences between control (215.80±10.60 a.u.) and AMPA (232.60±10.60 a.u.) treatment. For quantification of the synaptic

localization of $GABA_AR$-$\alpha 2$ in % see Fig. 6 t. n=3 with 16 cells per experiment. ANOVA was used for statistical analysis. Scale bar: 3 µm. Error bars: mean±s.e.m.

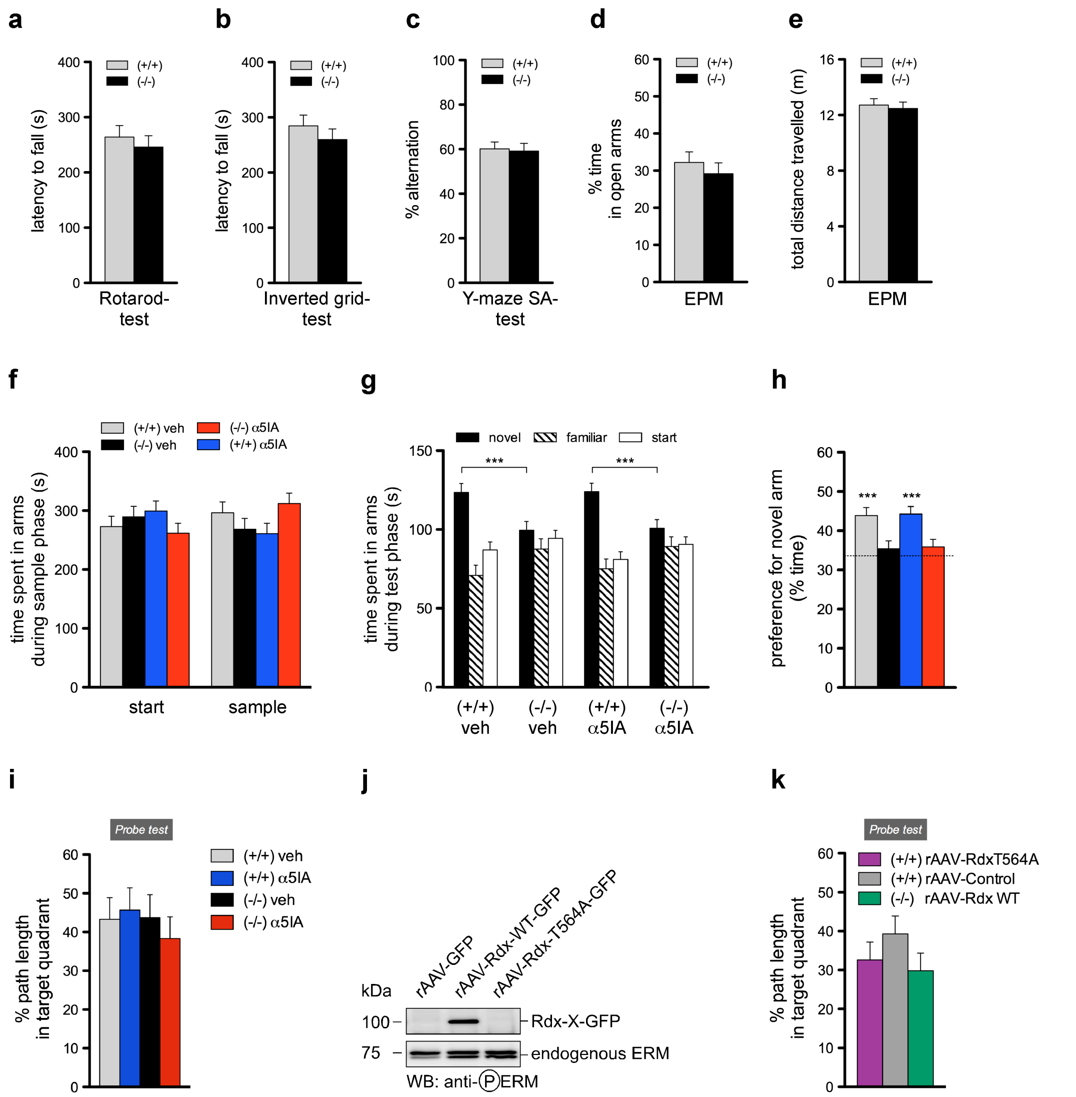

Supplementary Figure 7. Behavioural phenotyping of WT (+/+) and *Rdx*-KO (-/-) mice. (**a**) Rotarod test. The performance on the accelerating rotarod, indicated by the latency to fall off the rotating drum (max: 300 s), was comparable between WT (+/+) (264.25±18.79 s; *n*=8) and *Rdx*-KO (-/-) (245.88±22.44 s; *n*=8) mice (ANOVA; genotype $F_{1,14}$ = 0.394, P > 0.50). (**b**) Inverted grid test. The latency to fall off the inverted grid (hanging time after inversion; max: 300 s), was comparable between WT (+/+) (284.63±15.38 s; *n*=8) and *Rdx*-KO (-/-) (259.75±22.71 s; *n*=8) mice (ANOVA; genotype $F_{1,14}$ = 0.823, P > 0.35). Data presented in a and b indicates no impairment in motor function for Rdx-depleted mice. (**c**) Y-maze spontaneous alternation (SA) test. The analysis for percentage alternation [WT (+/+) *n*=14: 60.14±3.06 %; *Rdx*-KO (-/-) *n*=11: 59.18±3.46 %; ANOVA; $F_{1,23}$=0.043; P>0.80] revealed no differences between the genotypes. This indicates a normal alternation behavior for Rdx-depleted mice. (**d, e**) Elevated plus-maze (EPM) test. (d) Percentage time spent in the open arms and (e) total distance traveled was

comparable between *Rdx* (-/-) (*n*=28) and WT (+/+) (*n*=29) mice, indicating no differences in spontaneous anxiety-like behavior between the genotypes. (ANOVA: genotype $F_{1,55}$ =0.551, P > 0.5; $F_{1,55}$ = 0.147, P > 0.70, respectively). (**f-h**) Y-maze spatial recognition test with or without administration of a GABA$_A$Rα5 inverse agonist (α5IA). 30 min before sample phase, *Rdx*-KO (-/-) and WT (+/+) mice were injected (IP) with either α5IA or vehicle (veh). In the sample phase, mice were allowed to explore the start and a sample arm for 10 min. Access to the novel arm was blocked. Following a 1 h delay, the mice were examined for novelty preference (test phase) by allowing access and exploration to all three arms for 5 min. (**f**) The time spent in each arm during sample phase in the Y-maze spatial recognition test revealed no difference between (+/+)-veh (*n*=11), (-/-)-veh (*n*=11), (+/+)-α5IA (*n*=12) and (-/-)-α5IA (*n*=12) mice (ANOVA; arms x genotype: $F_{1,42}$ = 0.401, P > 0.5). This indicates comparable arm exploration [start arm: (+/+)-veh 272.99±17.73 s; (-/-)-veh 289.50±17.73 s; (+/+)-α5IA 299.41±16.98 s; (-/-)-α5IA 261.69±16.98 s; sample arm: (+/+)-veh 296.50±18.35 s; (-/-)-veh 268.54±18.35 s; (+/+)-α5IA 261.07±17.57 s; (-/-)-α5IA 312.22±17.57 s], independent of α5IA application (ANOVA; arms x genotype x drug: $F_{1,42}$ = 3.619, P > 0.05). (**g**) Mean time spent in all arms during novelty preference test (test phase). WT (+/+) mice spent significantly more time in the novel unfamiliar arm, compared to *Rdx* (-/-) mice (ANOVA; arms x genotype: $F_{2,84}$ = 9.116, P < 0.0001) independent of α5IA application (ANOVA; arms x genotype x drug: $F_{2,84}$ = 0.039, P > 0.90) [start arm: (+/+)-veh 87.10±5.11 s; (-/-)-veh 94.27±5.11 s ; (+/+)-α5IA 80.87±4.89 s; (-/-)-α5IA 90.54±4.89 s; familiar arm: (+/+)-veh 70.92±6.43 s; (-/-)-veh 87.71±6.43 s; (+/+)-α5IA 75.23±6.15 s; (-/-)-α5IA 89.21±6.15 s; novel arm: (+/+)-veh 123.43±5.69 s; (-/-)-veh 99.61±5.66 s; (+/+)-α5IA 123.93±5.42 s; (-/-)-α5IA 100.83±5.42 s]. (**h**) One-sample t-test against chance level performance (33%) revealed that preference for the novel arm in WT (+/+) mice was significantly above chance, whereas *Rdx* (-/-) mice performed at chance level, independent of α5IA application [(+/+)-veh 43.92±1.67 % t_{10}=6.35, P<0.0001; (-/-)-veh 35.43±2.19 % t_{10}=0.95, P>0.3; (+/+)-α5IA 44.25±2.03 % t_{11}=5.382, P<0.0001; (-/-)-α5IA 35.90±2.00 % t_{11}=1.285, P>0.2]. (**i**) Probe test following acquisition training in the MWM (shown in Fig. 7 j). Prior to the experiment, (+/+) and (-/-) mice were treated with or without α5IA. All groups showed a preference for the training quadrant [RM ANOVA: Main effect of quadrant ($F_{3,159}$ = 46.37, P < 0.0001) ((+/+)-veh 43.29±5.57 %; (+/+)-α5IA 45.67±5.76 %; (-/-)-veh 43.70±5.98 %; (-/-)-α5IA 38.32±5.57 %)]. (**j**) Western blot analysis of primary neurons 18 days after transduction with recombinant adeno-associated viruses (rAAV) as follows: rAAV-Rdx-WT-GFP, rAAV-RdxT564A-GFP or

rAAV-GFP control. Phosphorylated-ERM (PERM) protein levels were only detected for Rdx-WT-GFP, indicating an appropriate endogenous activation of the Rdx-WT-GFP fusion protein, which is sufficient for $GABA_AR$-$\alpha5$ binding. (**k**) Probe test following acquisition training in the MWM (shown in Fig. 8 c). Prior to the experiment, (+/+) and (-/-) mice [*Rdx* (+/+) rAAV-Rdx-T564A=7; *Rdx* (-/-) rAAV-Rdx-WT=7; *Rdx* (+/+) rAAV-Control=7] received bilateral hippocampal injections of rAAV-Rdx-X-GFP. All groups showed a preference for the training quadrant [RM ANOVA: Main effect of quadrant ($F_{3,54}$ = 7.80, P < 0.001) ((+/+) rAAV-RdxT564A 32.60±4.56 %; (+/+) rAAV-Control 39.32±4.56 %; (-/-) rAAV-Rdx-WT 29.81±4.56 %)]. Error bars: mean±s.e.m.

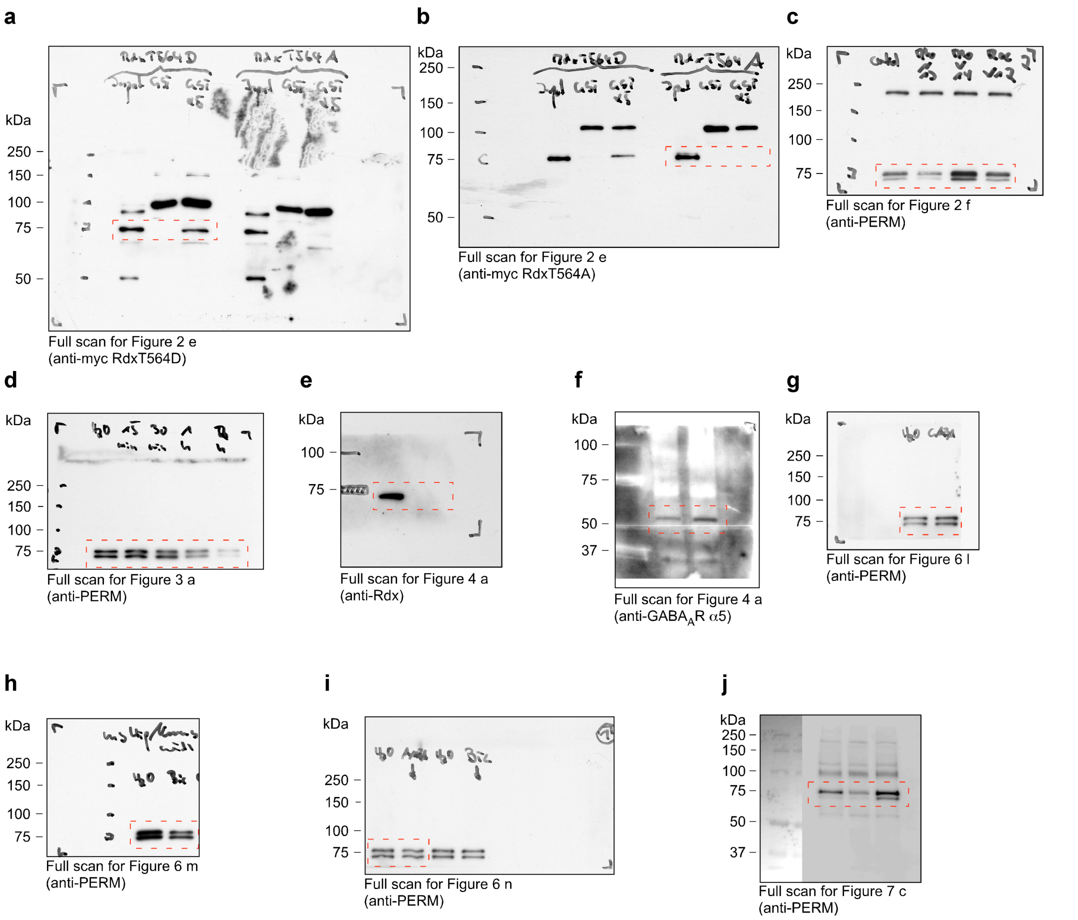

Supplementary Figure 8. Full scanned images of immunoblots presented in the main article. The cropped areas are outlined in dotted red lines. (**a-c**) Full scans for Fig. 2. (**d**) Full scan for Fig. 3. (**e-f**) Full scans for Fig. 4. (**g-i**) Full scans for Fig. 6. (**j**) Full scan for Fig. 7.

REFERENCES

1. Matsui T*, et al.* Rho-kinase phosphorylates COOH-terminal threonines of ezrin/radixin/moesin (ERM) proteins and regulates their head-to-tail association. *J Cell Biol* **140**, 647-657 (1998).

2. Klausner RD, Donaldson JG, Lippincott-Schwartz J. Brefeldin A: insights into the control of membrane traffic and organelle structure. *J Cell Biol* **116**, 1071-1080 (1992).

3. Chardin P, McCormick F. Brefeldin A: the advantage of being uncompetitive. *Cell* **97**, 153-155 (1999).

4. Lledo PM, Zhang X, Sudhof TC, Malenka RC, Nicoll RA. Postsynaptic membrane fusion and long-term potentiation. *Science* **279**, 399-403 (1998).

5. Jacob TC, Moss SJ, Jurd R. GABA(A) receptor trafficking and its role in the dynamic modulation of neuronal inhibition. *Nat Rev Neurosci* **9**, 331-343 (2008).

6. Rebola N, Pinheiro PC, Oliveira CR, Malva JO, Cunha RA. Subcellular localization of adenosine A(1) receptors in nerve terminals and synapses of the rat hippocampus. *Brain Res* **987**, 49-58 (2003).

7. Phillips GR*, et al.* The presynaptic particle web: ultrastructure, composition, dissolution, and reconstitution. *Neuron* **32**, 63-77 (2001).

8. Mortensen M, Kristiansen U, Ebert B, Frolund B, Krogsgaard-Larsen P, Smart TG. Activation of single heteromeric GABA(A) receptor ion channels by full and partial agonists. *J Physiol* **557**, 389-413 (2004).

9. Mortensen M, Ebert B, Wafford K, Smart TG. Distinct activities of GABA agonists at synaptic- and extrasynaptic-type GABAA receptors. *J Physiol* **588**, 1251-1268 (2010).
